# Levels of detail analysis of microwave scattering from human head models for brain stroke detection

**DOI:** 10.7717/peerj.4061

**Published:** 2017-11-21

**Authors:** Awais Munawar Qureshi, Zartasha Mustansar

**Affiliations:** Research Center for Modeling and Simulation (RCMS), National University of Sciences and Technology (NUST) H-12, Islamabad, Pakistan

**Keywords:** Microwave imaging, Finite element method, Human brain stroke, Forward problem, Inverse problem, Levels of detail

## Abstract

In this paper, we have presented a microwave scattering analysis from multiple human head models. This study incorporates different levels of detail in the human head models and its effect on microwave scattering phenomenon. Two levels of detail are taken into account; (i) Simplified ellipse shaped head model (ii) Anatomically realistic head model, implemented using 2-D geometry. In addition, heterogenic and frequency-dispersive behavior of the brain tissues has also been incorporated in our head models. It is identified during this study that the microwave scattering phenomenon changes significantly once the complexity of head model is increased by incorporating more details using magnetic resonance imaging database. It is also found out that the microwave scattering results match in both types of head model (i.e., geometrically simple and anatomically realistic), once the measurements are made in the structurally simplified regions. However, the results diverge considerably in the complex areas of brain due to the arbitrary shape interface of tissue layers in the anatomically realistic head model.

After incorporating various levels of detail, the solution of subject microwave scattering problem and the measurement of transmitted and backscattered signals were obtained using finite element method. Mesh convergence analysis was also performed to achieve error free results with a minimum number of mesh elements and a lesser degree of freedom in the fast computational time. The results were promising and the E-Field values converged for both simple and complex geometrical models. However, the E-Field difference between both types of head model at the same reference point differentiated a lot in terms of magnitude. At complex location, a high difference value of 0.04236 V/m was measured compared to the simple location, where it turned out to be 0.00197 V/m. This study also contributes to provide a comparison analysis between the direct and iterative solvers so as to find out the solution of subject microwave scattering problem in a minimum computational time along with memory resources requirement.

It is seen from this study that the microwave imaging may effectively be utilized for the detection, localization and differentiation of different types of brain stroke. The simulation results verified that the microwave imaging can be efficiently exploited to study the significant contrast between electric field values of the normal and abnormal brain tissues for the investigation of brain anomalies. In the end, a specific absorption rate analysis was carried out to compare the ionizing effects of microwave signals to different types of head model using a factor of safety for brain tissues. It is also suggested after careful study of various inversion methods in practice for microwave head imaging, that the contrast source inversion method may be more suitable and computationally efficient for such problems.

## Introduction

Over the years, microwave imaging (MWI) has been employed in various industrial and medical applications. The transmitted and reflected signals from the object-of-interest (OI) are measured and processed to construct reliable images of the target. MWI operating principle is based on significant contrast between the dielectric properties of the target and its surroundings (Pastorino, 2010). The Confocal Radar Technique (mono-static, bi-static or multi-static) and the Classical Inverse Scattering are two major approaches followed in active MWI (Jalilvand, Li & Zwick, 2013; Mohammed et al., 2015). In the radar approach, the backscattered signals are processed to indicate the location of significant scatterer (target). An inverse scattering approach utilizes the transmitted and backscattered fields’ information for solving an inverse scattering problem to construct shape of the target using spatial distribution of dielectric properties. In the medical field, an emerging diagnostic technique exploiting a Classical Inverse Scattering approach is known as microwave tomography (MWT). MWT relies on the considerable contrast between dielectric properties of the normal and abnormal tissues to indicate the area of disease.

Brain stroke is one of the leading causes of death in the world (Feigin, 2005; http://www.strokecenter.org/). A stroke interrupts the continuous supply of blood to the critical areas of brain. This phenomenon causes the denial of oxygen and nutrients to the brain tissues which eventually results in the loss of brain functions and death in many cases. Hemorrhagic and ischemic stroke are categorized as two major types of brain stroke. Sudden rupture of blood vessels causing bleeding in its surrounding areas is called a hemorrhagic stroke. In contrast, clotting in the blood arteries cause the blockage of blood circulation in brain and results in an ischemic stroke (SummitMedicalGroup, 2014). Both types of stroke exhibit some common symptoms such as; faintness, slurred speech, difficult swallowing and the sudden paralysis of body parts. However, an exclusive and timely medication is required for the treatment of each type of stroke from the onset of symptoms (Khan, Baguley & Cameron, 2003). This establishes the requirement of detecting and differentiating the type of brain stroke rapidly and reliably.

The present imaging techniques for brain stroke diagnostics include computed tomography (CT), magnetic resonance imaging (MRI) and positron emission tomography (PET). Although these techniques are quite efficient and provide a good resolution images of the brain but each has some associated constraints. They are all expensive, time-consuming and immobile imaging modalities. In particular, CT possesses ionizing effects, PET involves radioactive material injection and MRI is not suitable for the patients with metal biomedical implants (Beckmann, 2006; NPSMedicineWise, 2016; Ozomaro, 2013). Therefore, there is a need for an alternate imaging technique which can provide a safe, low-cost, portable and fast imaging solution for brain stroke detection. Thus, the MWT offers all these advantages along with non-ionizing and non-invasive features.

**Figure 1:**
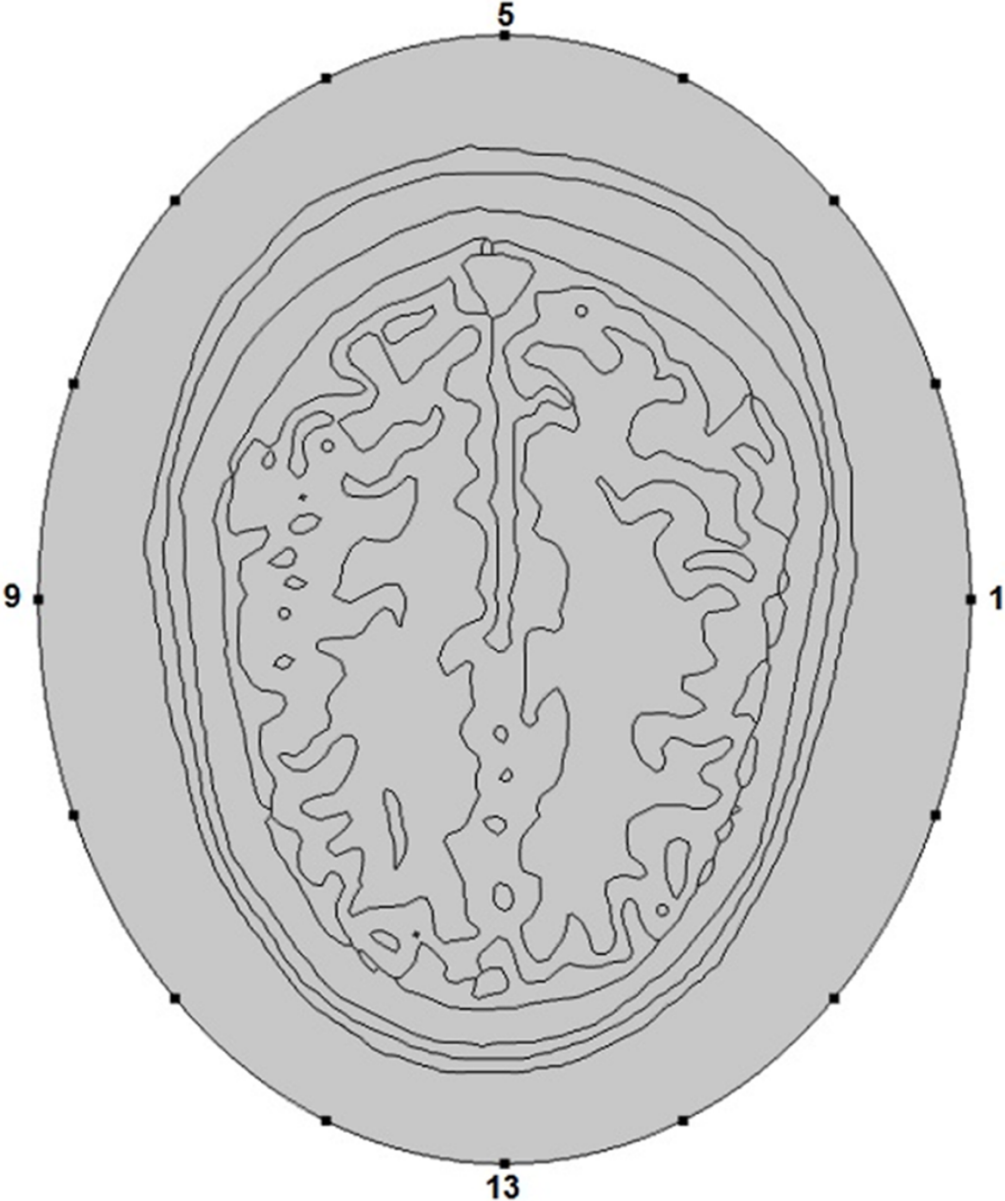
Layout of a microwave head imaging system.

Microwave head imaging can supplement existing brain imaging techniques (CT, MRI or PET) in emergency situations, especially at rural hospitals. It is suitable for continuous brain monitoring and can easily be deployed at emergency centers or first response paramedical ambulances. Microwave (MW) signals in the frequency range of 0.5–4.5 GHz and power level between 0 and 20 dBm, provide a reasonable compromise between the spatial resolution of brain images and the penetration of signals into human head (Mohammed et al., 2014). The layout of a conventional microwave head imaging setup comprises an array of transceivers, surrounding the human head and operating in a sequential mode at multi-frequency (Fig. 1).

The first study to assess the feasibility and performance potential of microwave imaging for brain stroke detection was conducted by Semenov & Corfield (2008). Semenov and co-worker performed computer simulations using a rudimentary 2-D circle based head model. The head model was comprised of five-layers of brain tissue with an emulated ischemic stroke of diameter 20 mm. A microwave signal between 0.5 and 2.0 GHz frequency at 20 dBm power level was utilized. In 2010, Ireland & Bialkowski (2010) performed a simulation study on the utilization of microwave signals’ penetration into the human head model for brain stroke detection. An anatomically realistic Zubal head phantom emulated with a hemorrhagic stroke of diameter 30 mm was involved in the simulations (Zubal et al., 1994). Later in 2011, the authors extended their previous work and developed a 2-D image reconstruction algorithm for brain stroke detection using microwave scattering information (Ireland & Bialkowski, 2011). A 2-D MRI slice of human head phantom, containing eight major types of brain tissue was selected. A Gaussian pulse signal (0.5–2.0 GHz, 20 dB SNR) and finite-difference time-domain (FDTD) numerical method were utilized to calculate the back-scattered electric field (E-Field) signals.

In 2010, Zakaria, Gilmore & LoVetri (2010) developed a 2-D image reconstruction algorithm for brain stroke detection based on a finite element method (FEM) and contrast source inversion (CSI) methodology. A 2-D ellipse based head model comprising five-layers of brain tissue with an emulated hemorrhagic stroke of diameter 20 mm was designed. This head model was irradiated by 1 GHz microwave signals using an array of 32 point sources, operating in a multi-static radar approach. Later on, Scapaticci et al. (2012) proposed design guidelines for the development of a microwave head imaging system to facilitate the maximum penetration of signals into human head and detect smaller size brain strokes. This design analysis involved a five-layer head model based on transmission line (TL) theory and incorporated frequency-dispersive behavior of brain tissues using single-pole Cole–Cole model. The authors suggested that the microwave signals in a frequency range of 0.6–1.5 GHz and a matching medium with dielectric properties of ε_mm_ = 40, σ_mm_ = 0.01 S/m are suitable for an effective head imaging setup. The proposed guidelines were duly validated through 2-D simulations using MRI slices, method of moment (MoM) numerical technique and a modified linear sampling method (LSM) based imaging strategy.

From 2013 onwards, remarkable development was made in the area of microwave head imaging for brain stroke analysis. In the same year, an analytical model for brain stroke detection using an ultra-wide band (UWB) radar approach was formulated by Jalilvand, Li & Zwick (2013). The 2-D planer head model comprised of five-layers of brain tissue with infinite length and an additional layer of blood (10 mm) to emulate the hemorrhagic stroke. UWB signals up to 10 GHz frequency with an effective power of 0 dBm at first layer of the head model were utilized to investigate the transmission and reflection of signals by each layer. Later on, Fhager et al. (2013) developed a microwave helmet-based head imaging setup for brain stroke detection and classification. An array of 10–12 triangular patch antennas operating in the frequency range of 0.1–3.0 GHz was mounted inside the helmet. A statistical classifier algorithm was also developed to differentiate a hemorrhagic stroke from the ischemic.

An approach similar to Fhager et al. (2013) was also followed by Mohammed et al. (2014) and Abbosh (2013) but utilizing an adjustable platform mounted with an array of 16 tapered slot antennas (1–4 GHz) and a fabricated head phantom comprising six-types of brain tissue (Mohammed et al., 2012a). A pre-processing technique was also applied to remove background reflections’ noise from the received signals (Mustafa, Mohammed & Abbosh, 2013). The high resolution images of phantom were constructed using a confocal delay-and-sum approach and the Fermat’s principle. Following this, the authors also performed computer simulations to investigate the possibility of utilizing a similar system for differentiating two types of brain stroke (Mobashsher et al., 2013; Mohammed, Abbosh & Ireland, 2012c). The SAM numerical head model assigned with average dielectric properties of brain tissues (ε_avg_ = 42, σ_avg_ = 0.99 S/m) was utilized. The brain stroke of different shapes (sphere, cylinder) and sizes (5∼20 mm radius) were emulated at various locations. The first approach was based on the comparison of reflection coefficients (S_11_) of an array of antennas (Mohammed, Abbosh & Ireland, 2012b). However, the second approach compared the reflection phases of two unidirectional antennas located symmetrically around the head model.

In the same year, (Priyadarshini & Rajkumar, 2013) performed computer simulations to study the electromagnetic (EM) waves scattering phenomenon by an ellipsoid head model for brain stroke analysis. The head model comprising four-layers of brain tissue with an emulated sphere shaped stroke of both types was utilized. The FEM numerical solver was deployed to compute the scattered EM fields by the head model at 1 GHz frequency. The dielectric profile of head model was reconstructed by solving an inverse scattering problem using CSI method. In 2014, Mobashsher & Abbosh (2014b), Mobashsher, Abbosh & Wang (2014) developed a portable microwave system for the detection of intracranial hemorrhage and traumatic brain injuries (TBIs). The system was designed using a customized transceiver and a unidirectional antenna (1.1–3.4 GHz) operating in a virtual array mono-static radar approach. The images of hemorrhagic affected head phantom were generated using techniques like noise removal and signal back-projection. The authors also fabricated a compact wideband directional antenna for microwave brain imaging applications utilizing the image theory on plane of symmetry (Mobashsher & Abbosh, 2014a).

Later in 2014, Jalilvand et al. (2014) presented a quantitative analysis of MWT using an anatomically realistic 2-D head model comprising five-types of brain tissue and an emulated hemorrhagic stroke (8.5 mm radius). An array of 24-point transceivers around the head model operated at 1 GHz frequency in a multi-static fashion. The FDTD numerical solver was utilized to solve a microwave scattering forward problem. An image reconstruction algorithm was developed following an iteratively optimized Gauss–Newton approach. A similar study was conducted by Bisio et al. (2016), however, the authors utilized MoM numerical technique to solve the forward problem. The inverse algorithm was based on the same iteratively regularized Gauss–Newton scheme. This study also incorporated the effect of background coupling medium permittivity on the results of image reconstruction algorithm.

In 2016, Mobashsher et al. (2016a), Mobashsher & Abbosh (2016) performed a design analysis and experimental evaluation of a portable microwave head imaging system for intracranial hemorrhagic detection (1–3 GHz). In addition, the authors also developed an improved back-projection algorithm to obtain reliable images of the brain (Mobashsher, Mahmoud & Abbosh, 2016b). Later on, Mohammed et al. (2016) proposed the replacement of co-axial cables with analogue fiber-optic links for connecting antenna array to the transceiver in a microwave head imaging system. The developed system was lightweight, compact and more efficient which resulted in an increased signal-to-noise ratio to construct an improved quality brain images. Multiple human head phantoms were also fabricated to conduct experimental studies on microwave head imaging (Mohammed & Abbosh, 2014; Otterskog, Petrovic & Risman, 2016). These phantoms reflected the realistic dielectric properties of main head tissues across MW frequency band with promising shelf life.

In the same year, a MoM–CSI methodology was implemented by Dilman et al. (2016) to evaluate the feasibility of microwave imaging for hemorrhagic stroke detection. This study utilized a 2-D realistic head model and introduced the noise effects along with the incorporation of different background mediums. Extending their previous work, in 2017 the authors adopted a differential MWI technique to monitor the progression of a hemorrhagic region over two time instants (Yıldırım et al., 2017). Later on, the compressive sensing and higher order basis functions were exploited by Stevanovic, Scapaticci & Crocco (2017), during the differential MWI of brain for stroke analysis. A principal component analysis (PCA)-based artefact removal technique and a modified delay-and-sum (DAS) beamforming algorithm were employed by Ricci et al. (2017) to perform the hemorrhagic stroke detection analysis on a 3-D simplified SAM head model.

Based on the literature discussed above, we already conducted an investigation study after careful literature survey to determine the feasibility of electromagnetic tomography (EMT) for brain stroke analysis, while making use of microwave signals (Munawar et al., 2016b). We performed computer simulations using a 3-D ellipsoid head model emulated with a hemorrhagic stroke. A half-wave dipole antenna operating in a transverse magnetic (TM) polarized mode at 1 GHz frequency was designed. The solution of a microwave scattering forward problem was found out by solving the Maxwell’s equations in time-harmonic form using FEM numerical solver. The simulation results were duly validated through an analytical solution of a 2-D multi-layer head model. Later on, we performed a comparison analysis of microwave scattering from a 2-D realistic head model for different types of brain stroke (Munawar, Mustansar & Maqsood, 2016a). The numerical head model was generated using MRI database and the dielectric properties were allocated to brain tissues accordingly. Sixteen point current sources were arranged in an elliptical array around the head model, operating in a sequential manner (1 mA, 1 GHz). It was demonstrated that the maximum E-Field difference exists at an approximate location of stroke with respect to normal brain tissues.

The study in this paper reveals different aspects of microwave scattering from human head models after incorporating the levels of detail analysis and a mesh convergence examination. Through image-based finite element simulations, we have explained the microwave scattering behavior of different complexity head models. Image-based modeling is a novel approach for the realistic simulation of models in a virtual reality (Wang et al., 2012; Young et al., 2008). The stack of 2-D images is converted into a high quality 3-D numerical model for subsequent use in engineering and biomedical simulations, while realizing the real-life environmental conditions. It is foreseen that the microwave scattering information may be effectively utilized during the development of an efficient image reconstruction algorithm for brain stroke detection, classification and progression monitoring.

In our current research, we have presented a microwave scattering comparison analysis between the geometrically simplified and the anatomically realistic head models. We have observed a significant change in the microwave scattering phenomenon as we increased the complexity of head models after making it anatomically more realistic. The literature available to-date, do not involve any dedicated study on such comparison. This is an important question to address, before solving the complex non-linear problems in brain stroke studies using MWT. We utilized FEM numerical solver to perform a mesh convergence analysis of the problem in hand to validate the reliability of generated meshes. Additionally, we have also conducted a comparison analysis between the direct and iterative solvers to find out the solution of subject 2-D microwave scattering problem in a time-efficient manner. This will help researchers working in the area of microwave brain imaging to make informed decisions for their future research.

## Methods

The development of a microwave head imaging system starts with the formulation of a microwave scattering problem in the simulation platform, while maintaining the real-time environmental conditions. It also requires modeling of a reliable and structurally detailed human head model. The forward problem of microwave scattering from head model is solved using an appropriate numerical technique. The solution of forward problem is utilized to develop an efficient image reconstruction algorithm to detect the presence and progression of brain strokes. The parametric settings of simulation setup provide design guidelines to develop an actual head imaging system. Different types of analysis are also performed to take into account the safety requirements of human head tissues against the exposure of microwave signals.

The possibility of extending 2-D scenario findings to 3-D microwave brain imaging applications was previously explored by Scapaticci et al. (2014). The authors worked on differential microwave imaging for brain stroke evolution monitoring to provide a proof-of-concept of the proposed follow-up procedure. In our current study, we have also described our research methodology using 2-D computer simulations. However, in future we aim to work in 3-D domain by extrapolating the results of our 2-D study to provide a more realistic and comprehensive analysis. In this section, we have explained our research methodology in a step-wise manner. Initially, we prepared a microwave scattering simulation setup for multiple human head models incorporated with frequency-dispersive dielectric properties. Later on, we utilized the solution to perform a mesh convergence and computational time analysis. The results from this section are fully capitalized in the subsequent simulations to validate our proposed methodology in an error free and time-efficient manner.

### Human head modeling

**Figure 2:**
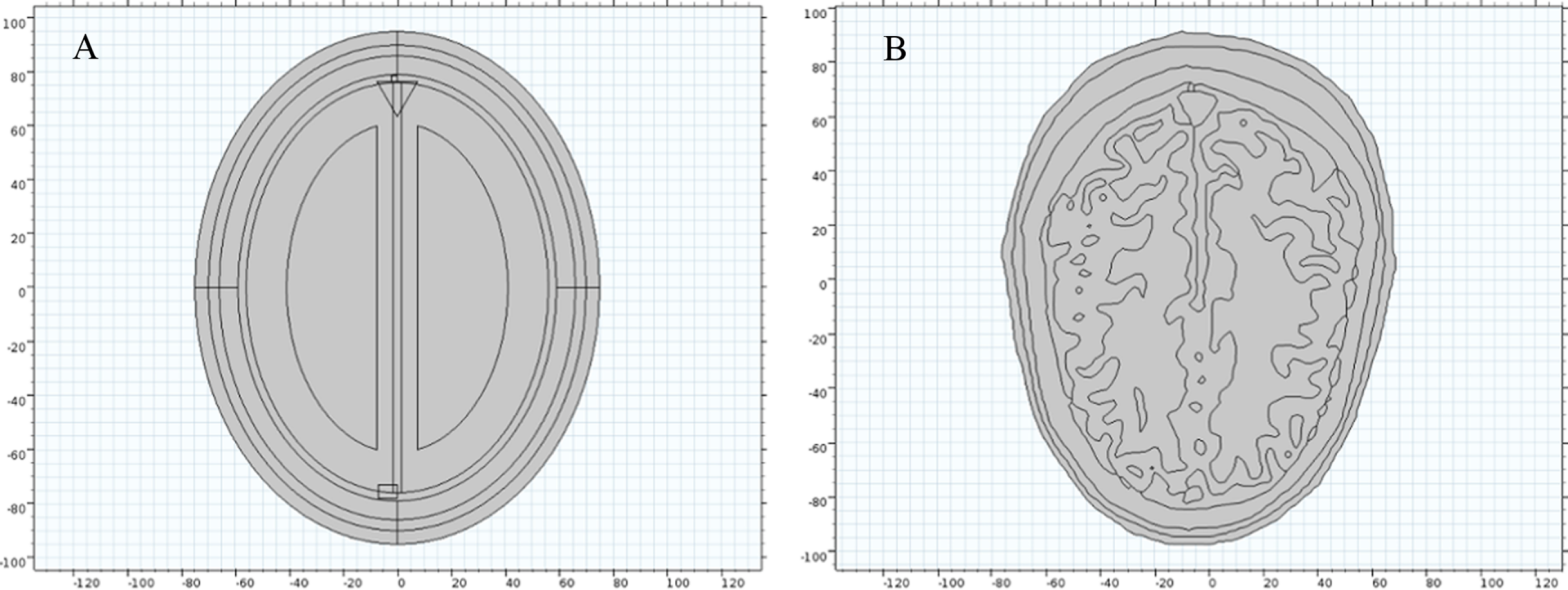
Human head models incorporated with different levels of detail. (A) Ellipse shaped head model. (B) Anatomically realistic head model.

In present 2-D simulation study, we have developed two types of human head model for performing microwave scattering analyses (Fig. 2). The first model is geometrically simplified and based on concentric ellipse shaped structure (Fig. 2A). It comprises of eight major layers of brain tissue; each built keeping in view the approximate radial thickness of actual MRI slice layers (Table 1). The second model is anatomically realistic and developed using the Zubal head phantom MRI database (Zubal, 1999). The MRI slices were imported in the Simpleware ScanIP image processing suite to create the numerical head model. The head tissues of different type were segmented using tissues identification list provided by the phantom developer. A 2-D geometrical model of the slice 36 was generated in an electromagnetic simulation environment from the Simpleware suite’s exported mesh in NASTRAN format (Fig. 2B).

**Table 1:** Geometrical dimensions and dielectric properties of the head tissues at 1 GHz frequency.

Tissue	Radial thickness (mm)	*X*-axis dia (mm)	*Y*-axis dia (mm)	Rel permittivity (ε_r_)	Conductivity σ (S/m)
Point source (air)	20	190	230	1	0
Skin (dry)	5	150	190	40.936	0.899770
Fat	4	140	180	5.447	0.053502
Skull (bone cortical)	7	132	172	12.363	0.155660
Cerebrospinal fluid (CSF)	3	118	158	68.439	2.455200
Gray matter (GM)	15	112	152	52.282	0.985410
White matter (WM)	33.5	82	122	38.577	0.621900
Dura	15	–	–	44.201	0.993300
Hemorrhagic (blood)	–	20	20	61.065	1.582900
Ischemic (clot)	–	20	20	30	0.5

**Figure 3:**
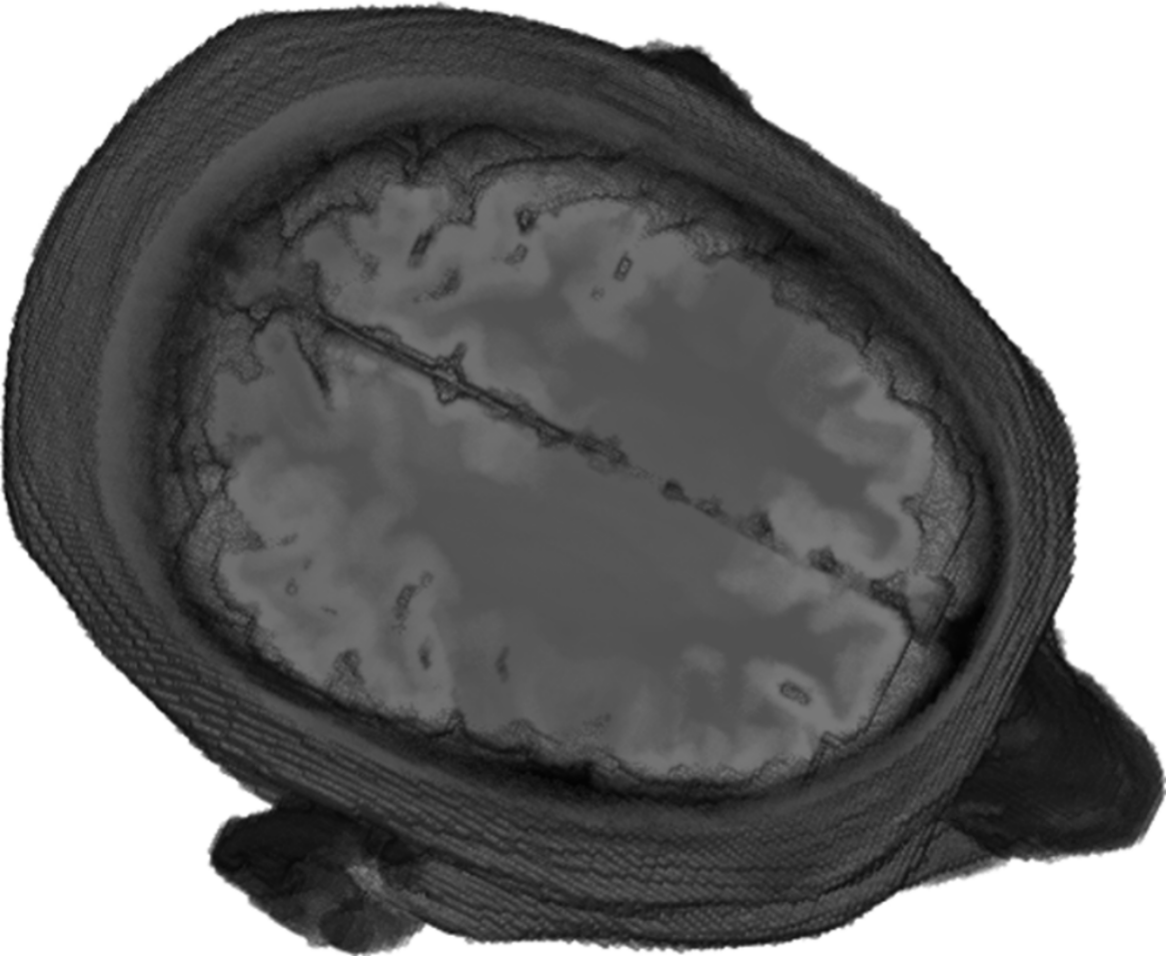
A 3-D cross-sectional view of the Zubal head phantom.

The Zubal head phantom comprises of 256 × 256 × 128 cubical elements, where the size of each voxel is 1.1 × 1.1 × 1.4 mm. A 3-D cross-sectional view of the Zubal head phantom is shown in Fig. 3. In our 2-D simulation studies, we have selected slice 36 of the Zubal head phantom so that we could construct the geometry of ellipse shaped head model with matching anatomy, where each model comprises of eight major types of head tissue. In addition, we could compare our simulation results with previous studies involving slice 36 of the Zubal head phantom (Ireland & Abbosh, 2013; Ireland, Bialkowski & Abbosh, 2013; Ireland & Bialkowski, 2011; Mohammed et al., 2015; Mustafa, Abbosh & Nguyen, 2014; Scapaticci et al., 2012). The forward problem modeling of microwave scattering also involves the incorporation of heterogenic and frequency-dispersive behavior of brain tissues in the numerical head models. The dielectric properties (ε; permittivity and σ; conductivity) to different types of head tissues are assigned using multiple frequency-dependent closed-form equations.

### Dielectric properties assignment

**Figure 4:**
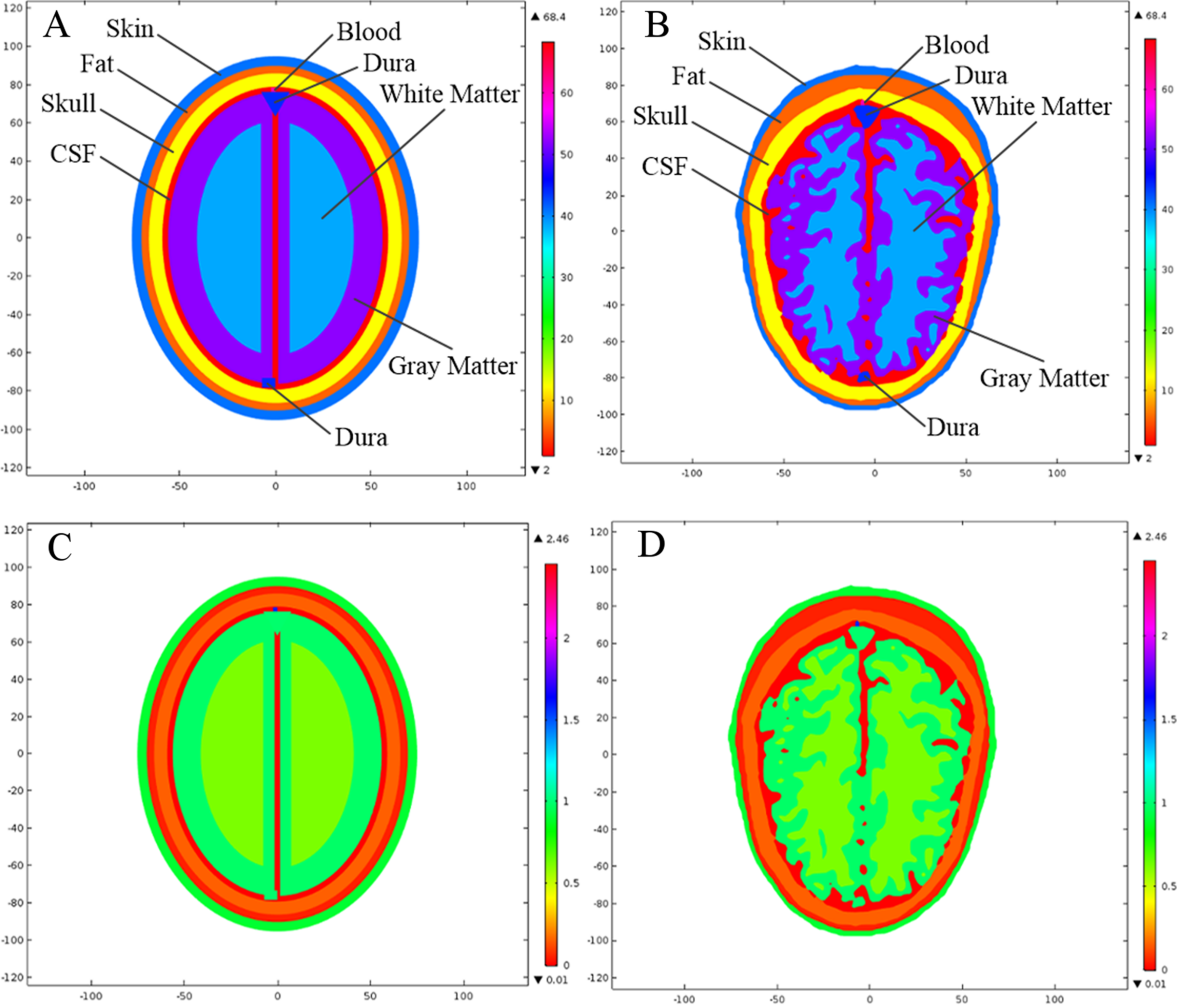
Dielectric profile of the human head models at 1 GHz frequency. (A) Relative permittivity of the ellipse head model. (B) Relative permittivity of the realistic head model. (C) Conductivity of the ellipse head model. (D) Conductivity of the realistic head model.

In our simulations, we have allocated dielectric properties to different types of head tissue using fourth-order Cole–Cole frequency-dependent model (Gabriel, 1996; Gabriel, Gabriel & Corthout, 1996a; Gabriel, Lau & Gabriel, 1996b, 1996c). An online application based on this model was also developed by Andreuccetti, Fossi & Petrucci (2002). This model is valid for the assignment of dielectric properties to various types of human body tissue across 10 Hz to 20 GHz frequency range. It is highlighted that the most suitable frequency reported in the literature for microwave head imaging is 1 GHz. This frequency offers a reasonable compromise between the spatial resolution of brain images and the penetration of microwave signals into human head. Therefore, the frequency-dispersive model is evaluated at 1 GHz and the simulation results have been presented at the same frequency. Table 1 lists the dielectric properties of eight major types of head tissue at 1 GHz frequency, including the hemorrhagic and ischemic stroke respectively. Figure 4 shows the relative permittivity (ε_r_) and conductivity (σ) assignment to both types of head model at 1 GHz frequency.

### Forward problem modeling

**Figure 5:**
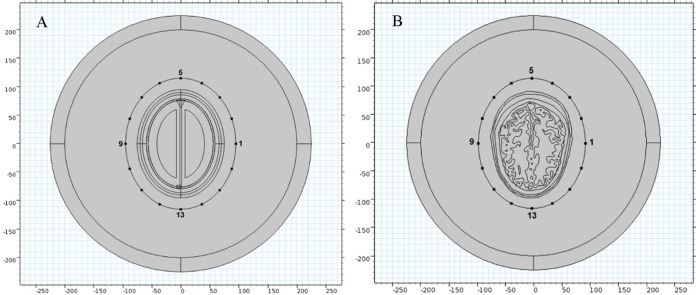
Forward problem setup. (A) Ellipse head model. (B) Realistic head model.

The forward problem modeling and analysis guides us in finalizing the design of a microwave head imaging system. The solution of forward problem helps in determining an appropriate placement of microwave transceivers, a suitable frequency range and the allowable power level. In our 2-D forward problem simulations, we have utilized sixteen point current sources arranged in an elliptical array around each head model. They are placed at a 2–3 cm distance from the side of head model with equidistant separation among them. Each point current source operates at 1 mA current and transmits a TM-polarized signal at 1 GHz frequency in a sequential manner. In order to realize the real-time environmental conditions, the computational domain around the head model is truncated using perfectly matched layer (PML) boundary condition. Moreover, the continuity boundary conditions have been applied between different layers of the brain tissue as well as the air and head model (Eq. 1), where *E* is the electric field intensity [V/m]. Figure 5 shows the forward problem setup for both types of head model used in our simulation studies.

(1) $$\hskip7\hat \hskip-6pt{\bi n} \times \left( {{{\vec {\bi E}}_1} - {{\vec {\bi E}}_2}} \right) = 0$$

The solution of above modeled forward problem is found out to investigate the interaction between microwave signals and different types of head model. Electric field values at numerous locations inside and around the head models are calculated using Maxwell’s equations. Based upon the application in hand, an appropriate numerical method is employed to find out the solution of Maxwell’s equations in time or frequency domain. FDTD, MoM, FEM and boundary element method (BEM) are few examples (Davidson, 2005; Sadiku, 2011). In our simulations, we have calculated the electric field values of transmitted and backscattered microwave signals utilizing the Helmholtz wave equation (Eq. 2), derived from Maxwell’s equations in time-harmonic form, where *E* is the electric field intensity [V/m], *μ*_r_ is the relative permeability [H/m], *ε*_r_ is the relative permittivity [F/m], ${\rm k_0} = \omega \sqrt {{\mu _0}{\varepsilon _0}} $ is the free-space wave number [m^−1^], ω is the angular frequency [rad/s] and σ is the electric conductivity [S/m].

(2) $$\nabla \times \mu _{\rm r}^{ - 1}\left( {\nabla \times \vec {\bi E}} \right) - k_0^2\left( {{\varepsilon _{\rm{r}}} - {{j\rsigma } \over {\omega {\varepsilon _0}}}} \right)\vec {\bi E} = 0$$

**Figure 6:**
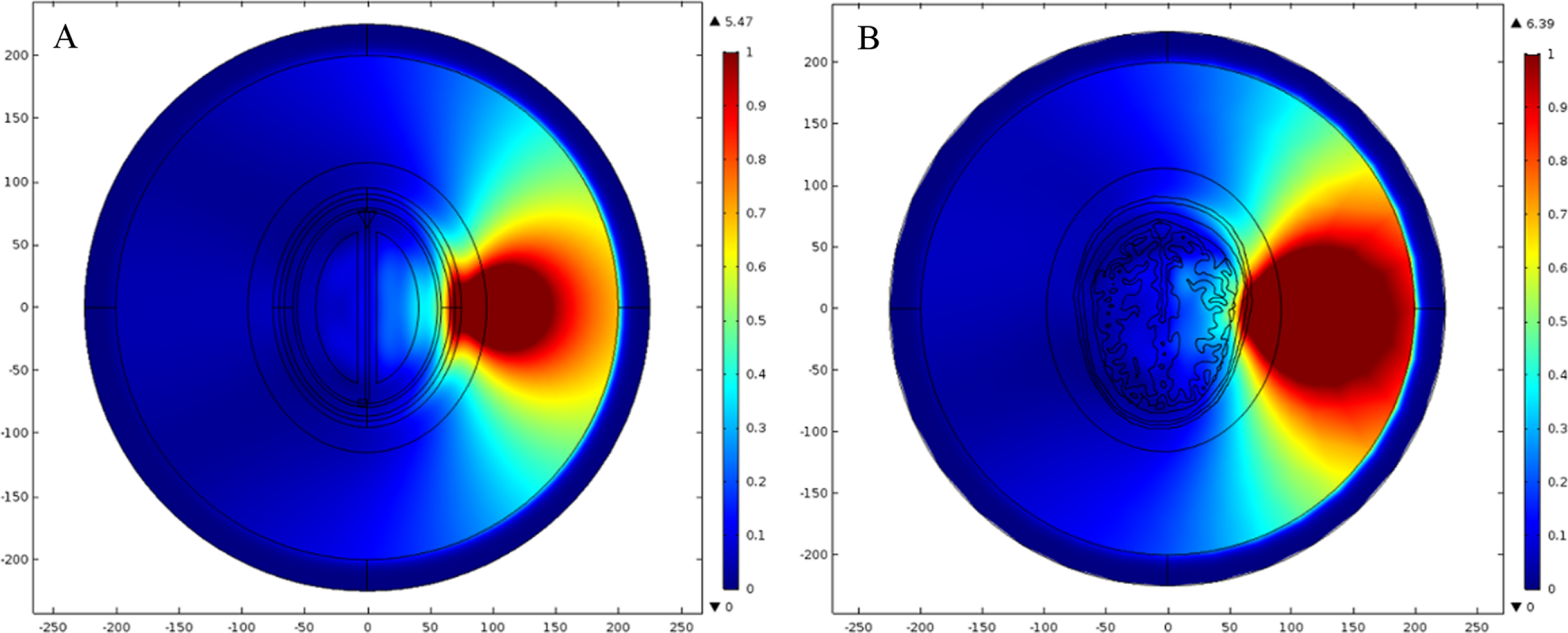
Spatial distribution of E-Norm at 1 GHz frequency using Pt Src1. (A) Ellipse head model. (B) Realistic head model.

We have applied FEM numerical technique to find out the solution of our forward problem models in frequency domain. FEM is selected due to its ability to model complex geometries and deal with anisotropic dielectric materials with least discretization errors in a non-homogeneous background medium. FEM is also preferred over FDTD or MoM numerical methods for performing a steady-state analysis of microwave scattering problems. Figure 6 shows the spatial distribution of electric field norm (E-Norm) inside both types of head model at 1 GHz frequency using point source 1. In figure, the color bar represents E-Norm value (V/m) with red color indicating the locations of higher E-Norm values and the blue color highlights the lower ones. In the case of the ellipse shaped head model, the FE mesh comprised 5,094 domain elements and the solution converged in 6 s with 37,323 number degrees of freedom (DOF). However, the FE mesh for realistic head model comprised 30,128 domain elements and the solution converged in 17 s with 212,561 number degrees of freedom.

### Brain stroke emulation

**Figure 7:**
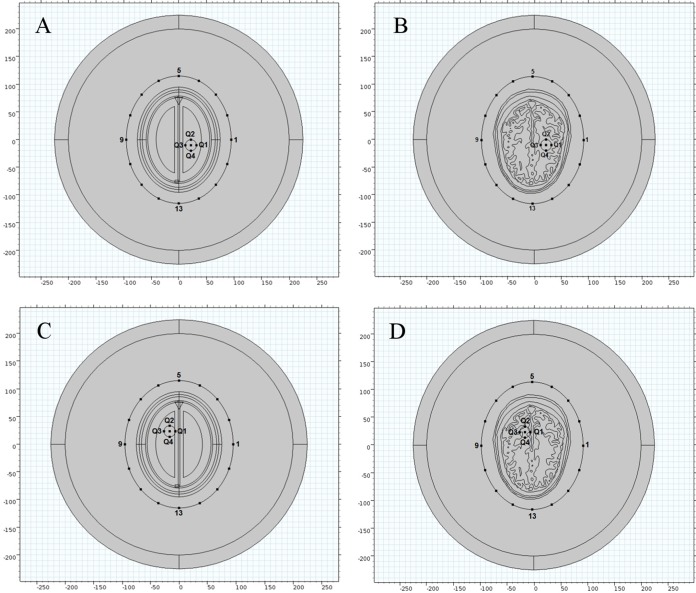
Locations of an emulated brain stroke inside the head models. (A) Ellipse head model location 1. (B) Realistic head model location 1. (C) Ellipse head model location 2. (D) Realistic head model location 2.

**Figure 8:**
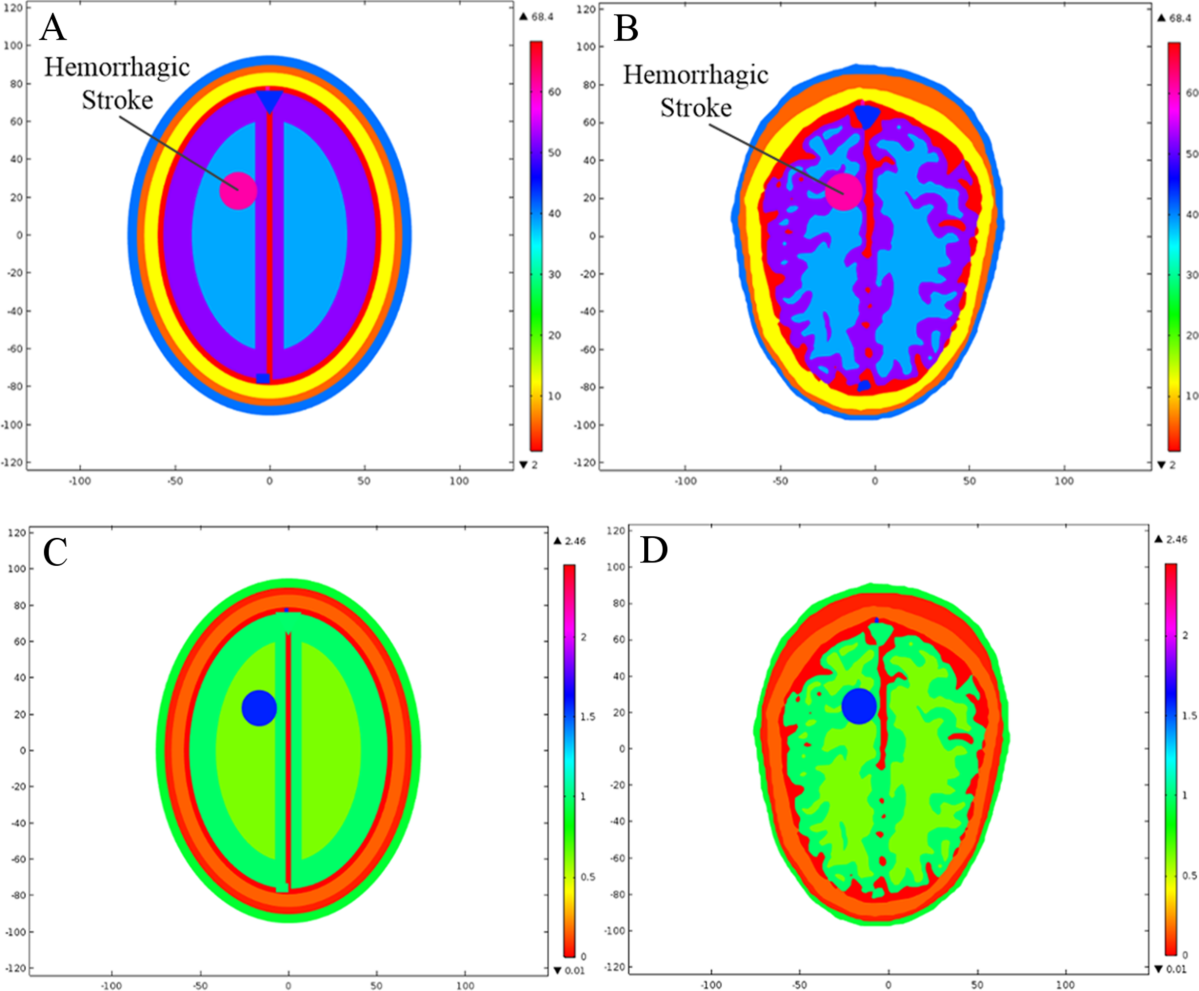
Dielectric profile of the hemorrhagic affected head models at 1 GHz frequency and stroke location 2. (A) Relative permittivity of the ellipse head model. (B) Relative permittivity of the realistic head model. (C) Conductivity of the ellipse head model. (D) Conductivity of the realistic head model.

In order to analyze the effects of brain stroke on microwave scattering phenomenon, a circle shaped stroke with a diameter of 20 mm was emulated at different locations inside the head models. The first location was selected in an anatomically simpler region of the realistic head model, with more analogy to the ellipse layered head model. The stroke was centered at a point (22, −10 mm) in both types of head model. The second location was selected in a complex region of the realistic head model, with less similarity to the ellipse layered head model. The stroke was centered at a point (−16.5, 23.5 mm) in both types of head model. Figure 7 shows the two different locations of an emulated stroke inside both types of head model. Two main types of brain stroke were investigated at both locations inside the head models. The hemorrhagic stroke was assigned dielectric properties of (ε_r_ = 61.0650, σ = 1.5829), whereas the ischemic stroke was having dielectric properties of (ε_r_ = 30, σ = 0.5) at 1 GHz frequency. Figure 8 maps the dielectric profile of both types of head model at 1 GHz frequency with an emulated hemorrhagic stroke at location 2.

### Mesh convergence analysis

Mesh convergence analysis has been performed in this study for a human head microwave scattering problem using FEM numerical solver. The aim was to achieve error free results with the least number of mesh elements and a reduced number of degrees of freedom. The solution was converged regardless of the mesh size or density in a minimum computational time. In order to have a better comparison analysis between the normal and stroke affected head models, we kept the mesh size same during each case. Therefore, the emulated stroke circle was filled with healthy brain tissues’ dielectric properties in the normal case. During FE mesh generation, the criterion of at least five mesh elements per wavelength (λ/5) was considered so that the solution of subject electromagnetic problem converged accurately.

The simulations were run on a high performance workstation with an Intel Core i7-5500 processor (2.4 GHz) and 16 GB RAM. A mesh convergence analysis was performed for the stroke area only in both types of head model using E-Norm evaluation. An error tolerance of 1e^−3^ was assumed during the convergence of E-Norm value. The brain stroke emulated at a complex location 2 but filled with healthy tissues dielectric properties and the point source 7 facing the stroke circle were utilized. We employed the PARDISO direct method to find out the solution of subject 2-D microwave scattering problem during the mesh convergence analysis.

**Figure 9:**
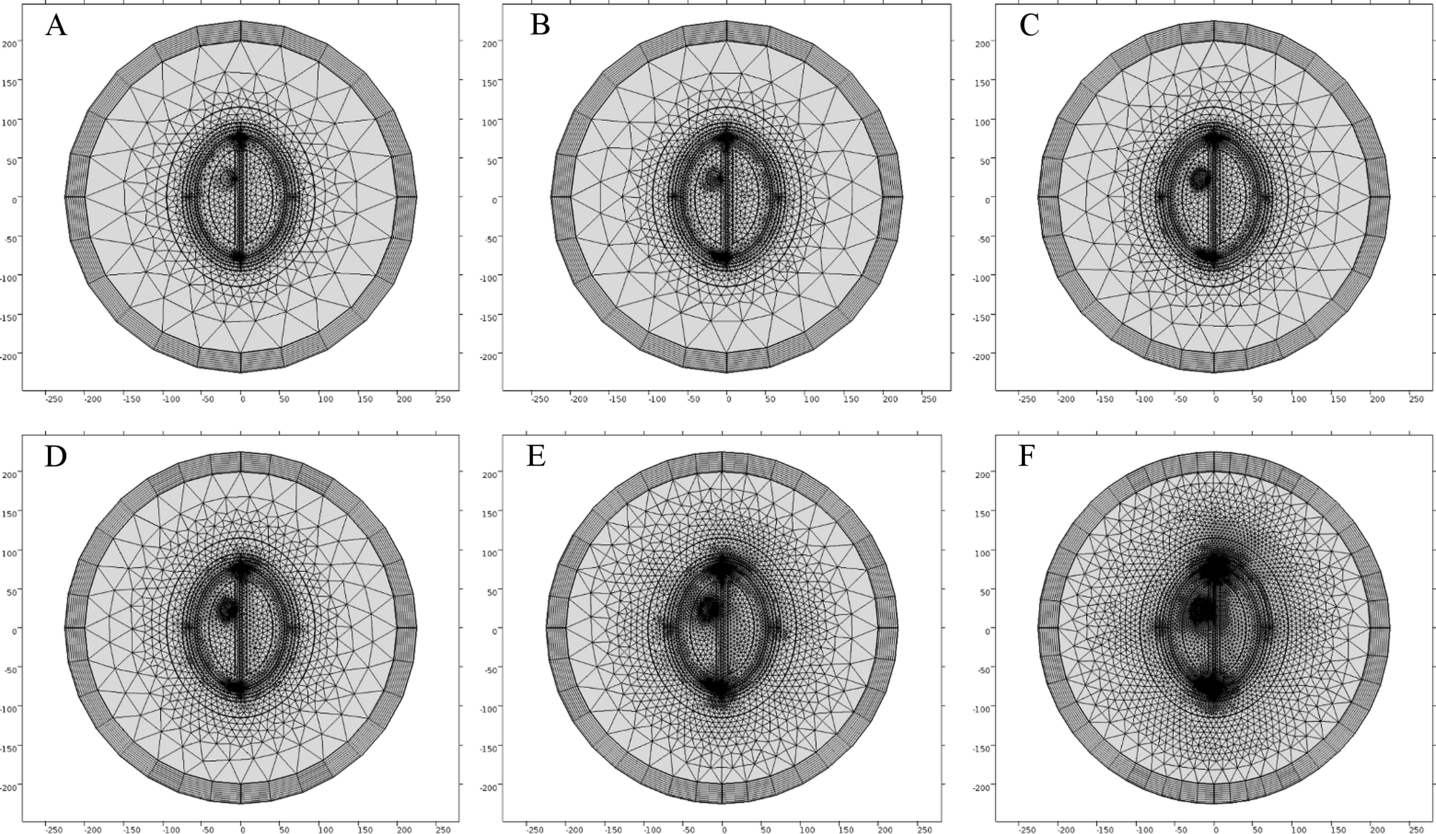
FE mesh mapping onto the ellipse head model. (A) Coarser mesh. (B) Coarse mesh. (C) Normal mesh. (D) Finer mesh. (E) Extra fine. (F) Extremely fine.

**Figure 10:**
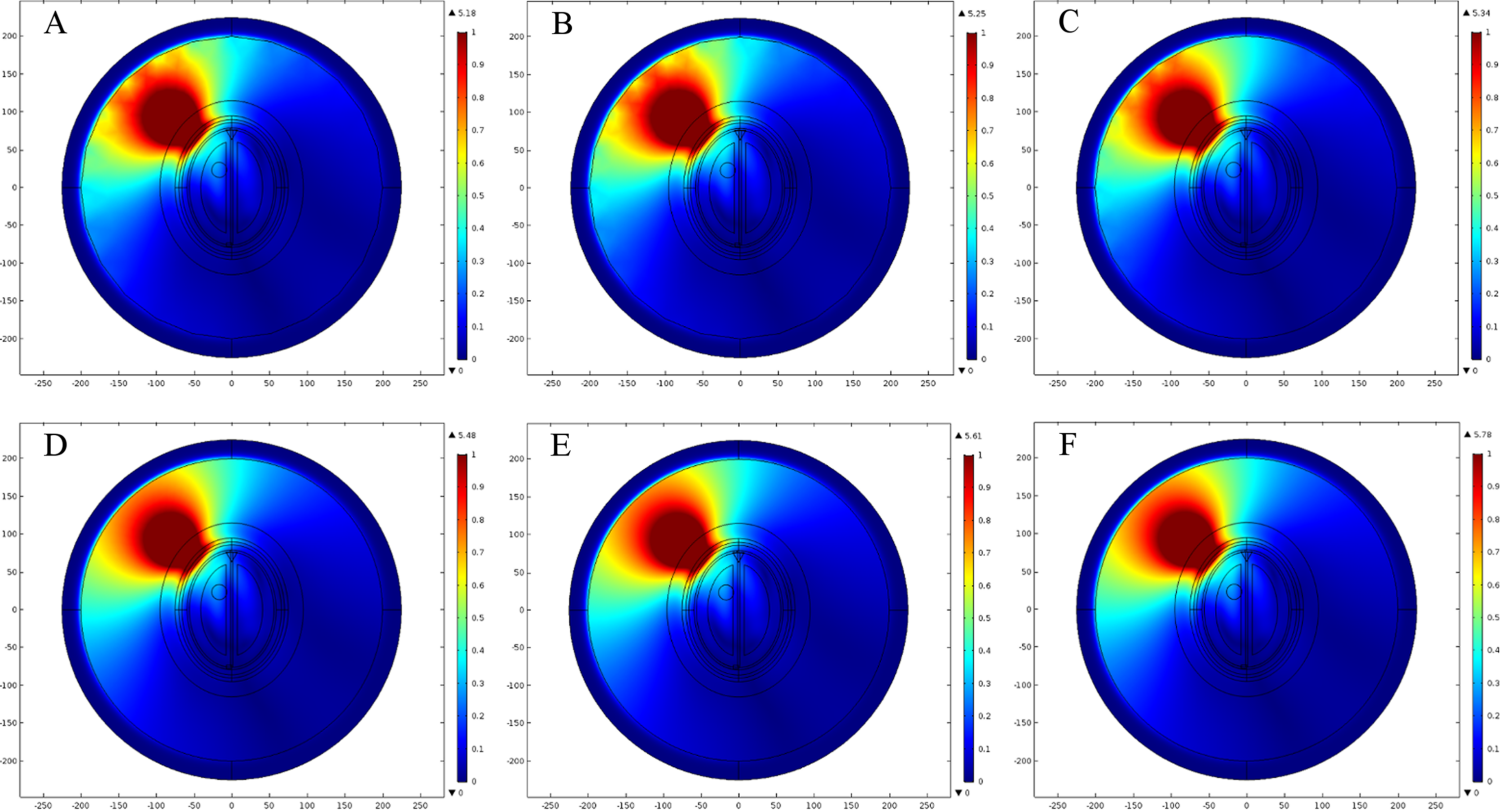
Spatial distribution of E-Norm inside the ellipse head model at 1 GHz frequency using Pt Src7. (A) Coarser mesh. (B) Coarse mesh. (C) Normal mesh. (D) Finer mesh. (E) Extra fine. (F) Extremely fine.

**Figure 11:**
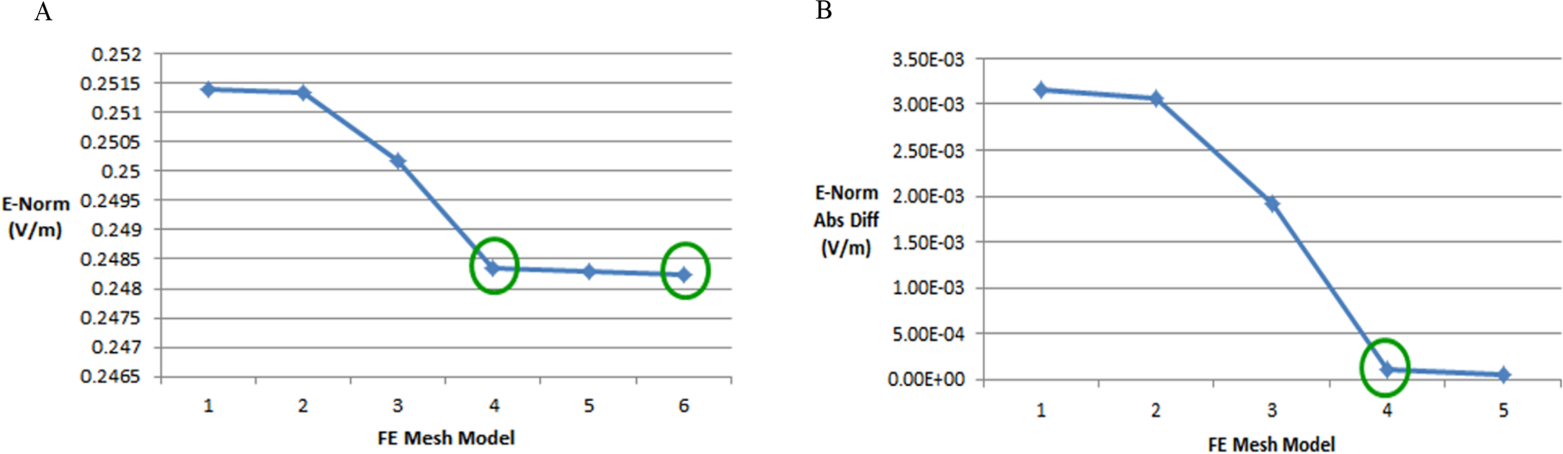
FE mesh convergence analysis for the ellipse head model. (A) E-Norm evaluation at the center of stroke circle. (B) E-Norm absolute difference wrt extremely fine mesh.

We generated six types of FE mesh model with different level of coarseness for the ellipse head model (Table 2). Figure 9 shows the mesh mapping of each FE model onto the ellipse head model and Fig. 10 displays the spatial distribution of E-Norm against each type of mesh model. The information about solution time, number of degrees of freedom and E-Norm evaluation at the center of stroke circle is provided in Table 3. It was inferred from the convergence analysis of E-Norm value that FE model 4 (Finer) provided a reliable solution within the tolerance error of 1e^−3^ once compared to FE Model 6 results (Extremely Fine). Moreover, FE Model 4 comprised half the number of mesh elements without inverted ones and provided the solution quickly with a lesser number of degrees of freedom. Therefore, FE Model 4 was preferred over the finer FE Models (5 and 6) and utilized during our subsequent levels of detail analysis of microwave scattering from the ellipse head model. Figure 11 shows the FE mesh convergence analysis for the ellipse head model, based on E-Norm and E-Norm absolute difference evaluation at the center of stroke circle.

**Table 2:** FE mesh models with element size parameters for the ellipse head model.

Model no.	Mesh type	Max size (mm)	Min size (mm)	Elements	Inverted elements	Max element growth rate	Curvature factor
1	Coarser	59.96	0.2174	3,860	24	1.5	0.6
2	Coarse	59.96	0.2174	4,506	24	1.35	0.4
3	Normal	59.96	0.135	4,996	20	1.3	0.3
4	Finer	59.96	0.0563	5,594	Nil	1.25	0.25
5	Extra fine	59.96	0.0337	7,898	Nil	1.15	0.25
6	Extremely fine	59.96	0.009	11,590	Nil	1.1	0.2

**Table 3:** FE solution information and E-Norm evaluation at the center of stroke circle against different mesh models for the ellipse head model.

Model no.	Mesh type	Solution time (s)	DOF	E-Norm (V/m)	E-Norm abs diff (V/m)
1	Coarser	5	28,269	0.2514	3.15E-03
2	Coarse	6	32,791	0.25132	3.07E-03
3	Normal	6	36,429	0.25017	1.92E-03
4	Finer	6	40,823	0.24836	1.10E-04
5	Extra fine	7	57,159	0.24830	5.00E-05
6	Extremely fine	10	83,627	0.24825	–

**Figure 12:**
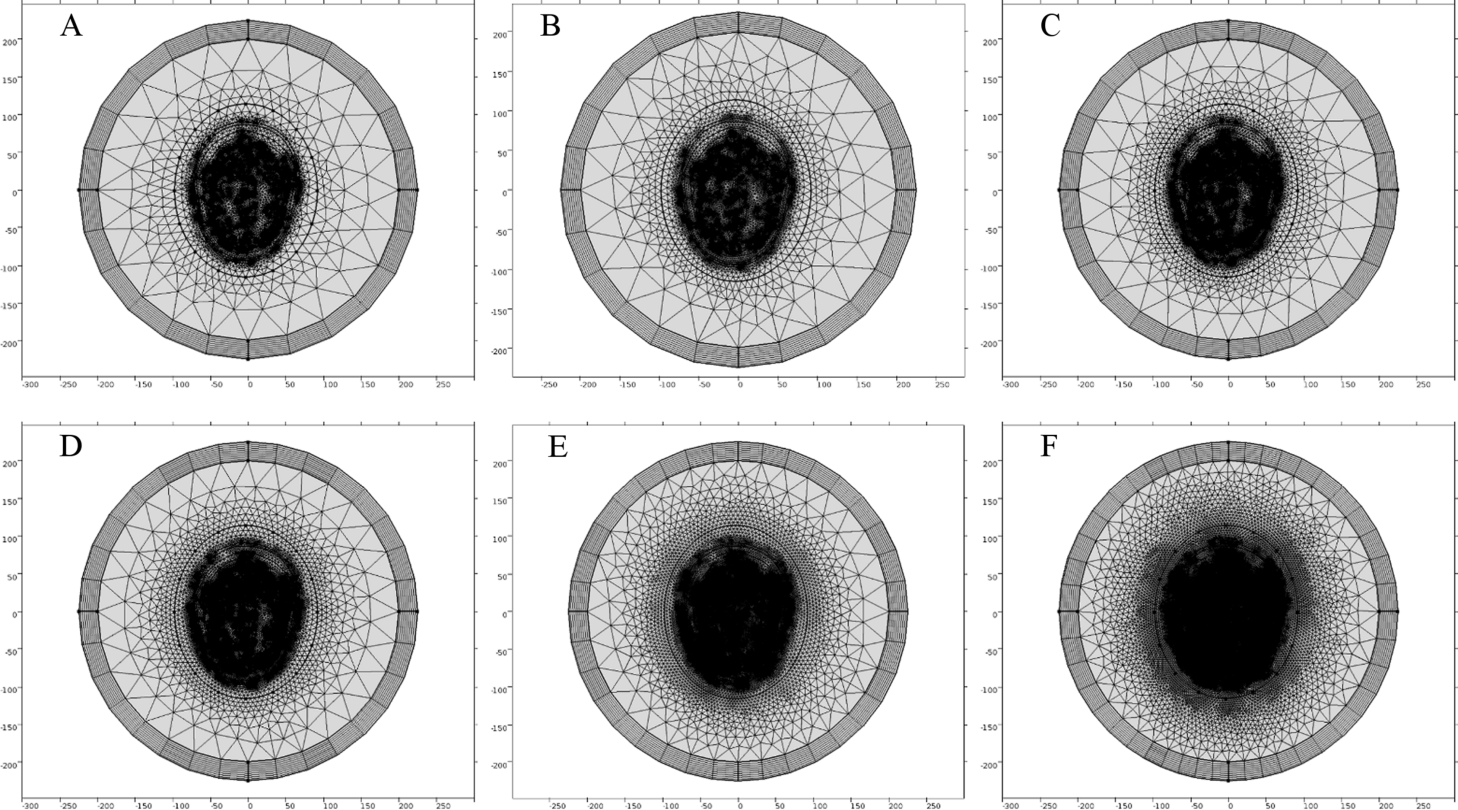
FE mesh mapping onto the realistic head model. (A) Coarser mesh. (B) Coarse mesh. (C) Normal mesh. (D) Finer mesh. (E) Extra fine. (F) Extremely fine.

**Figure 13:**
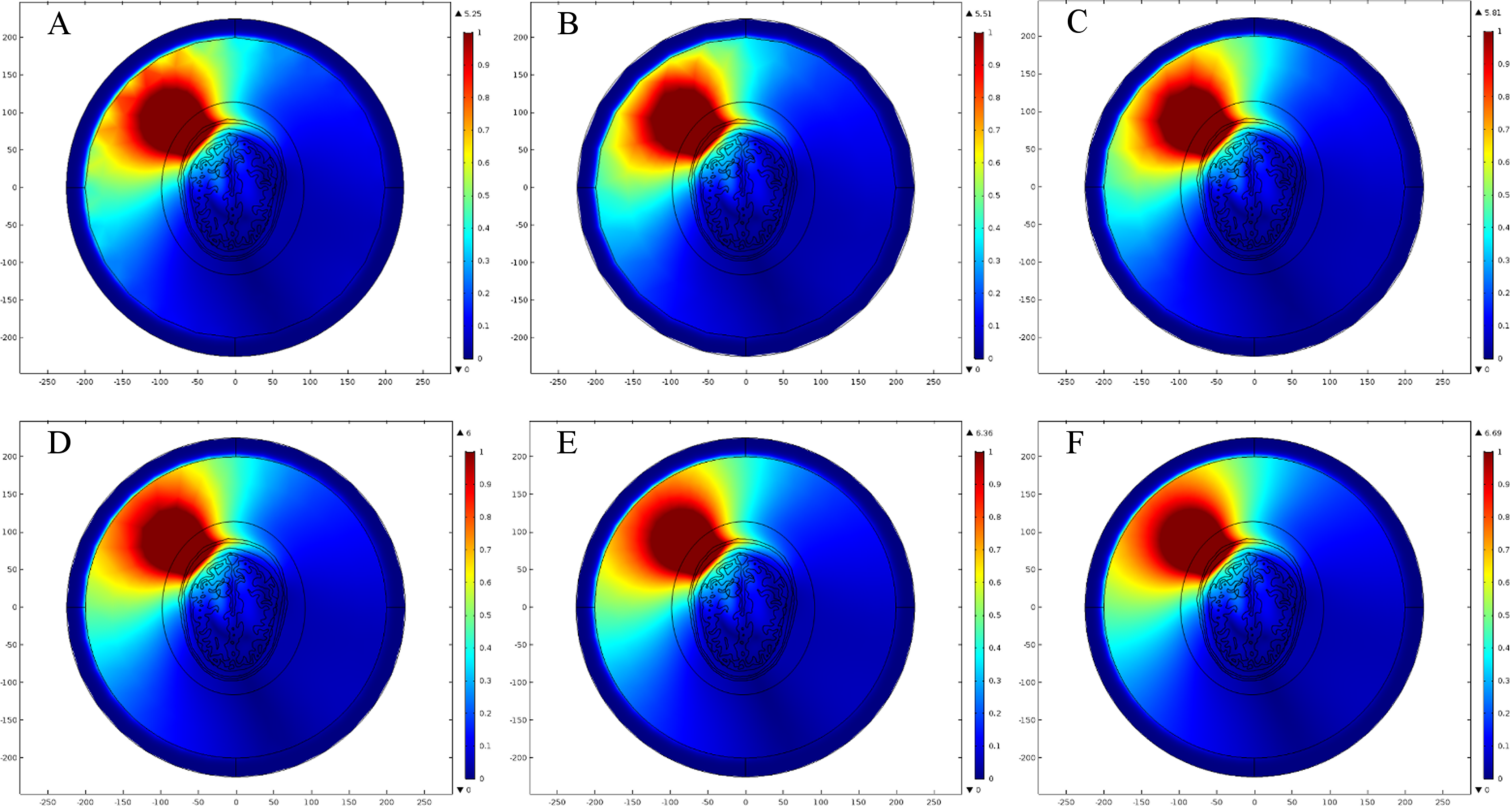
Spatial distribution of E-Norm inside the realistic head model at 1 GHz frequency using Pt Src7. (A) Coarser mesh. (B) Coarse mesh. (C) Normal mesh. (D) Finer mesh. (E) Extra fine. (F) Extremely fine.

**Figure 14:**
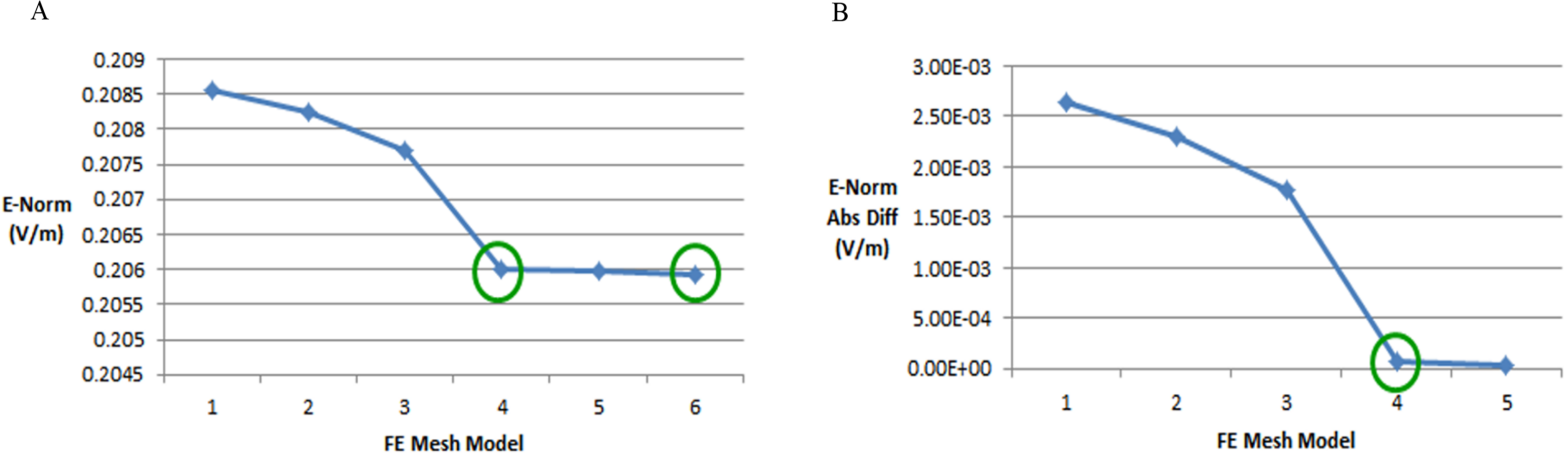
FE mesh convergence analysis for the realistic head model. (A) E-Norm evaluation at the center of stroke circle. (B) E-Norm absolute difference wrt extremely fine mesh.

In order to determine an efficient FE mesh for the realistic head model, we performed a similar type of mesh convergence study. Six types of FE mesh model were generated for the anatomically realistic head model as well (Table 4). The mesh mapping of each FE model onto the realistic head model is shown in Fig. 12 and the spatial distribution of E-Norm against each type of mesh model are portrayed in Fig. 13. Table 5 provides the details of solution time, number of degrees of freedom and E-Norm value at the center of stroke circle. During E-Norm value convergence analysis, it was figured out that FE model 4 (Finer) offered an acceptable solution with an error of 1e^−3^ once compared to FE Model 6 results (Extremely Fine). In addition, FE model 4 was less than a half dense and solved the subject microwave scattering problem in a time-efficient manner with a reduced number of degrees of freedom. Therefore, we preferred FE Model 4 over the finer FE Models (5 and 6) for performing microwave scattering analysis of the realistic head model. Figure 14 shows the FE mesh convergence analysis for the realistic head model, based on E-Norm and E-Norm absolute difference evaluation at the center of stroke circle.

**Table 4:** FE mesh models with element size parameters for the realistic head model.

Model no.	Mesh type	Max size (mm)	Min size (mm)	Elements	Inverted elements	Max element growth rate	Curvature factor
1	Coarser	59.96	0.2174	19,904	24	1.5	0.6
2	Coarse	59.96	0.2174	25,042	24	1.35	0.4
3	Normal	59.96	0.135	28,630	20	1.3	0.3
4	Finer	59.96	0.0563	32,988	Nil	1.25	0.25
5	Extra fine	59.96	0.0337	50,626	Nil	1.15	0.25
6	Extremely fine	59.96	0.009	76,172	Nil	1.1	0.2

**Table 5:** FE solution information and E-Norm evaluation at the center of stroke circle against different mesh models for the realistic head model.

Model no.	Mesh type	Solution time (s)	DOF	E-Norm (V/m)	E-Norm abs diff (V/m)
1	Coarser	11	140,577	0.20856	2.63E-03
2	Coarse	13	176,543	0.20823	2.30E-03
3	Normal	16	201,867	0.2077	1.77E-03
4	Finer	17	232,581	0.206	7.00E-05
5	Extra fine	25	356,255	0.20597	4.00E-05
6	Extremely fine	35	535,597	0.20593	–

### Computational time analysis

In order to solve the subject microwave scattering problem in a time-efficient manner, we performed a comparison analysis between the direct and iterative solvers for each type of head model. Three types of direct solver were studied: multifrontal massively parallel sparse (MUMPS), parallel direct sparse solver (PARDISO) and sparse object oriented linear equations solver (SPOOLS). All were based on lower–upper (LU) decomposition of the sparse matrices. We also considered three types of iterative solver: generalized minimum residual (GMRES), flexible generalized minimum residual (FGMRES) and Biconjugate gradient stabilized (BiCGStab) with Multigrid option. A microwave scattering problem was solved for both types of head model using each solver type. The FE mesh Model 4 (Finer), the point source 7 and the stroke circle at location 2 but filled with normal brain tissues dielectric properties were utilized.

The details of computational time and the memory resources occupied by each solver while finding out the solution of a microwave scattering problem for the ellipse head model are given in Table 6. Similar details are provided in Table 7 but using the anatomically realistic head model. It has been observed that in the case of the direct solvers, PARDISO method found out the solution in a minimum computational time for both types of head model. However, in the case of the iterative solvers, FGMRES method outperformed the other iterative methods for each head model. It is also noteworthy to mention that each direct and iterative solver utilized approximately the same memory resources while finding out the forward problem solution for a particular head model. Since we were dealing with a 2-D electromagnetic problem, we preferred the use of PARDISO direct solver to perform our subsequent levels of detail analysis in a time and memory efficient manner once compared with FGMRES iterative solver.

**Table 6:** Computational time and memory resources utilization of the microwave scattering problem for the ellipse head model.

Solver type		Solution time (s)	Iterations	Physical memory (GB)	Virtual memory (GB)
**Direct solver**	MUMPS	5	1	1.16	1.55
	PARDISO	4	1	1.16	1.55
	SPOOLS	7	1	1.12	1.51
**Iterative solver**	GMRES	11	80	1.14	1.53
	FGMRES	9	72	1.15	1.54
	BiCGStab	14	48	1.16	1.55

**Table 7:** Computational time and memory resources utilization of the microwave scattering problem for the realistic head model.

Solver type		Solution time (s)	Iterations	Physical memory (GB)	Virtual memory (GB)
**Direct solver**	MUMPS	15	1	1.33	1.94
	PARDISO	12	1	1.38	2.00
	SPOOLS	27	1	1.4	1.98
**Iterative solver**	GMRES	45	64	1.42	2.03
	FGMRES	34	73	1.39	1.94
	BiCGStab	62	48	1.33	1.89

## Results and Discussion

In this section, we have demonstrated that how the microwave scattering phenomenon changed significantly as we incorporated more levels of detail in the human head models during our computer simulations. We have shown that a considerable contrast between E-Field values exists at an approximate location of stroke affected tissues once compared to the normal brain. The importance of 2-D modeling and the prospective extension of 2-D simulation results to a 3-D scenario have been discussed briefly. It is also explained in this study that the microwave scattering information may be effectively utilized during the development of an image reconstruction algorithm for brain stroke detection and differentiation. Therefore, we have proposed a suitable imaging strategy based upon a careful literature survey. In addition, an analysis has been performed to determine the safety of microwave signals for human head imaging applications.

### E-Field distribution analysis

The present study considered three cases (Normal, Hemorrhagic and Ischemic) to compare the microwave scattering phenomenon between two types of human head model: (i) Simplified ellipse shaped, (ii) Anatomically realistic. The brain stroke effects were analyzed at two different locations: (i) Simple region, (ii) Complex region. The point source 1 was considered for a simple location 1, whereas the point source 7 was utilized for a complex location 2. Both point sources were placed at a 2–3 cm distance from the side of head models and transmitted a TM-polarized signal at 1 GHz frequency. The FE mesh Model 4 (Finer) and the PARDISO direct solver were utilized for both head models to perform these computer simulations. We selected the center of stroke circle as a reference point to perform the E-Field distribution comparison analysis. The center point was preferred because it could reflect meaningful changes in the E-Norm values, present due to the stroke affected tissues dielectric properties and the microwave signals’ transmission/reflection phenomenon.

#### Normal brain

**Figure 15:**
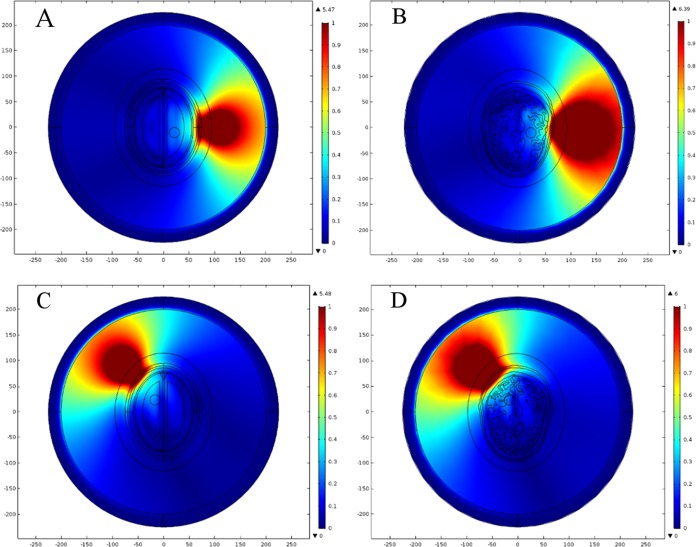
Spatial distribution of E-Norm inside the normal head models at 1 GHz frequency. (A) Ellipse head model using Pt Src1. (B) Realistic head model using Pt Src1. (C) Ellipse head model using Pt Src7. (D) Realistic head model using Pt Src7.

For the normal brain E-Field distribution comparison analysis, the emulated stroke circle was filled with a healthy brain tissues’ dielectric properties in both types of head model. Since the stroke circle for a simple location 1 was created in the white matter (WM) region, therefore it was filled with WM tissues dielectric properties (WM: ε_r_ = 38.577, σ = 0.6219 S/m). However, the stroke circle for a complex location 2 was created across the white and gray matter (GM) regions, therefore it was filled with corresponding region dielectric properties (GM: ε_r_ = 52.282, σ = 0.98541 S/m). Figure 15 shows the spatial distribution of E-Norm inside the normal head models for two different point sources at 1 GHz frequency. Table 8 provides the details of E-Norm absolute difference between the normal head models evaluated at five points of the stroke circle, created at two different locations. It has been observed that an anatomically realistic head model exhibits more microwave scattering phenomenon once compared with a geometrically simplified ellipse head model. In the case of the ellipse head model, the penetration of E-Field is more progressive and uniform as it encounters the tissue layers of different dielectric properties. By contrast, in the case of the realistic head model the E-Field penetration is less due to the non-uniform and complex structure of the head model.

**Table 8:** E-Norm absolute difference (V/m) between the normal head models evaluated at stroke circle points for two different locations.

Stroke location	Head model	*Q*3 (V/m)	*Q*4 (V/m)	Center (V/m)	*Q*2 (V/m)	*Q*1 (V/m)
Pt Src					
**Simple loc1** **Pt Src1**	Ellipse model	0.19556	0.21699	0.22545	0.22885	0.27305
	Realistic model	0.18699	0.23623	0.22742	0.22481	0.29296
	Abs diff (V/m)	0.00857	0.01924	0.00197	0.00404	0.01991
**Complex loc2** **Pt Src7**	Ellipse model	0.26592	0.2185	0.24836	0.2812	0.135
	Realistic model	0.2675	0.2026	0.206	0.2442	0.10706
	Abs diff (V/m)	0.00158	0.0159	0.04236	0.037	0.02794

In the case of a simple location 1, the E-Norm absolute difference between both head models at the center of stroke circle was calculated as 0.00197 V/m, whereas the maximum difference 0.01991 V/m existed at Q1 point. Similarly in the case of a complex location 2, the E-Norm absolute difference between both head models at the center of stroke circle was calculated as 0.04236 V/m and the same was maximum as well. It has been observed that the E-Norm absolute difference between both head models at the center of stroke circle is greater at a complex location 2 once compared to the simple location 1. It is due to the fact that arbitrarily shaped structure of a complex region in the realistic head model contrasts a lot with respect to an ellipse shaped head model. As a result, a complex region demonstrates more microwave scattering phenomenon and the greater E-Norm absolute differences are observed. Therefore, it is recommended to use an anatomically realistic human head model while performing the computer simulations, so as to realize the actual behavior of different layers of brain tissue.

#### Hemorrhagic affected brain

**Figure 16:**
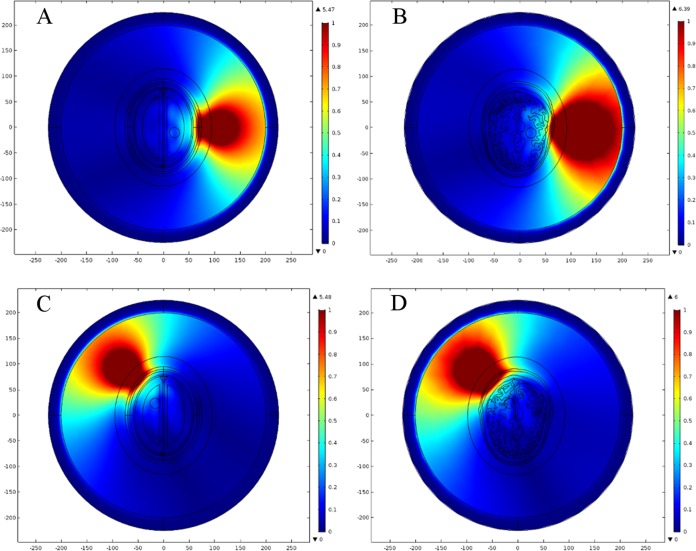
Spatial distribution of E-Norm inside the hemorrhagic affected head models at 1 GHz frequency. (A) Ellipse head model using Pt Src1. (B) Realistic head model using Pt Src1. (C) Ellipse head model using Pt Src7. (D) Realistic head model using Pt Src7.

In order to perform the E-Field distribution comparison analysis for the hemorrhagic affected head models, the stroke circle was filled with blood dielectric properties (ε_r_ = 61.065, σ = 1.5829 S/m) to emulate the bleeding caused by the ruptured blood vessels. Figure 16 shows the spatial distribution of E-Norm inside the hemorrhagic affected head models for two different point sources at 1 GHz frequency. Table 9 provides the details of E-Norm absolute difference between the hemorrhagic affected head models evaluated at five points of the stroke circle, created at two different locations. In the case of the hemorrhagic stroke emulated at a simple location 1, the E-Norm absolute difference between both head models at the center of stroke circle was calculated as 0.00413 V/m, whereas the maximum difference 0.02532 V/m existed at Q4 point. Similarly in the case of the hemorrhagic stroke emulated at a complex location 2, the E-Norm absolute difference between both head models at the center of stroke circle was calculated as 0.02359 V/m, whereas the maximum difference 0.02468 V/m existed at Q2 point. Similar to the normal brain case, the greater E-Norm absolute difference between both head models at the center of stroke circle was observed once the hemorrhagic stroke was emulated at a complex location 2 as compared to the simple location 1.

**Table 9:** E-Norm absolute difference (V/m) between the hemorrhagic affected head models evaluated at stroke circle points for two different locations.

Stroke location	Head model	*Q*3 (V/m)	*Q*4 (V/m)	Center (V/m)	*Q*2 (V/m)	*Q*1 (V/m)
Pt Src					
**Simple loc1** **Pt Src1**	Ellipse model	0.19288	0.1622	0.17665	0.17558	0.26013
	Realistic model	0.19083	0.18752	0.18078	0.1751	0.26876
	Abs diff (V/m)	0.00205	0.02532	0.00413	0.00048	0.00863
**Complex Loc2** **Pt Src7**	Ellipse model	0.21712	0.1883	0.19805	0.2382	0.11828
	Realistic model	0.23094	0.18641	0.17446	0.21352	0.10702
	Abs diff (V/m)	0.01382	0.00189	0.02359	0.02468	0.01126

#### Ischemic affected brain

**Figure 17:**
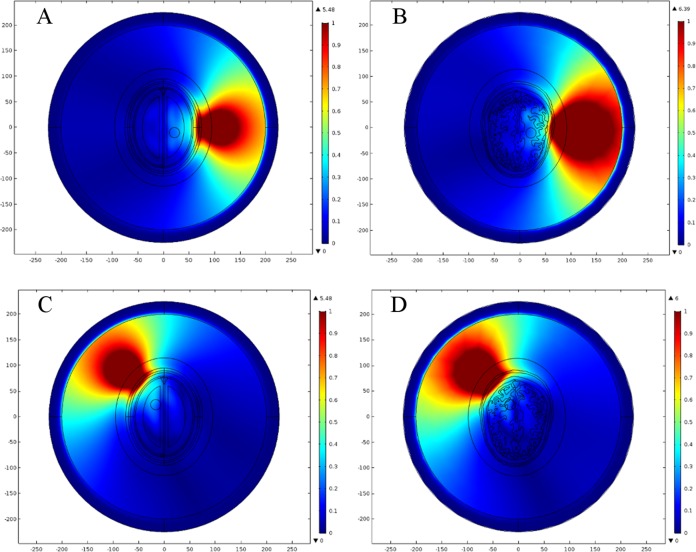
Spatial distribution of E-Norm inside the ischemic affected head models at 1 GHz frequency. (A) Ellipse head model using Pt Src1. (B) Realistic head model using Pt Src1. (C) Ellipse head model using Pt Src7. (D) Realistic head model using Pt Src7.

In the case of E-Field distribution comparison analysis for the ischemic affected head models, the stroke circle was filled with embolus dielectric properties (ε_r_ = 30, σ = 0.5 S/m) to emulate the blockage caused by the clotted blood arteries. Figure 17 shows the spatial distribution of E-Norm inside the ischemic affected head models for two different point sources at 1 GHz frequency. Table 10 provides the details of E-Norm absolute difference between the ischemic affected head models evaluated at five points of the stroke circle, created at two different locations. In the case of the ischemic stroke emulated at a simple location 1, the E-Norm absolute difference between both head models at the center of stroke circle was calculated as 0.0006 V/m, whereas the maximum difference 0.02109 V/m existed at Q1 point. Similarly in the case of the ischemic stroke emulated at a complex location 2, the E-Norm absolute difference between both head models at the center of stroke circle was calculated as 0.03863 V/m and the same was maximum as well. Similar to the above two cases, the greater E-Norm absolute difference between both head models at the center of stroke circle was observed once the ischemic stroke was emulated at a complex location 2 once compared with the simple location 1.

**Table 10:** E-Norm absolute difference (V/m) between the ischemic affected head models evaluated at stroke circle points for two different locations.

Stroke location	Head model	*Q*3 (V/m)	*Q*4 (V/m)	Center (V/m)	*Q*2 (V/m)	*Q*1 (V/m)
Pt Src					
**Simple loc1** **Pt Src1**	Ellipse model	0.17654	0.22568	0.22256	0.23974	0.2879
	Realistic model	0.16554	0.2454	0.22316	0.22969	0.30899
	Abs diff (V/m)	0.011	0.01972	0.0006	0.01005	0.02109
**Complex loc2** **Pt Src7**	Ellipse model	0.2858	0.20782	0.24334	0.29997	0.12383
	Realistic model	0.28831	0.19039	0.20471	0.26137	0.090462
	Abs diff (V/m)	0.00251	0.01743	0.03863	0.0386	0.033368

### E-Norm absolute difference analysis

Through computer simulations, we have demonstrated that the microwave imaging may be effectively utilized for the detection and differentiation of different types of brain stroke. There exists a significant contrast between E-Field values of the normal and stroke affected brain tissues. We have calculated the E-Norm absolute difference values to identify the location and types of brain stroke during our microwave scattering forward problem simulations. E-Norm absolute difference is defined as the absolute value of the difference between E-Field Norm evaluated at same point in both head models (i.e., normal and stroke affected). In addition, these E-Field differences may be efficiently utilized to find out the solution of an inverse scattering problem during the development of an image reconstruction algorithm. In real-time, the developed image reconstruction algorithm exploits only the transmitted and scattered E-Field signals information to find out the dielectric properties of different layers of human brain. Based upon these calculations, the brain images are constructed to accurately locate and classify the types of brain stroke, present due to abnormal tissues’ dielectric properties. We have emulated two types of brain stroke at two different locations inside both head models to explain the changes in E-Field distribution of the stroke affected brain models with respect to normal case.

#### Brain stroke at simple location 1

**Figure 18:**
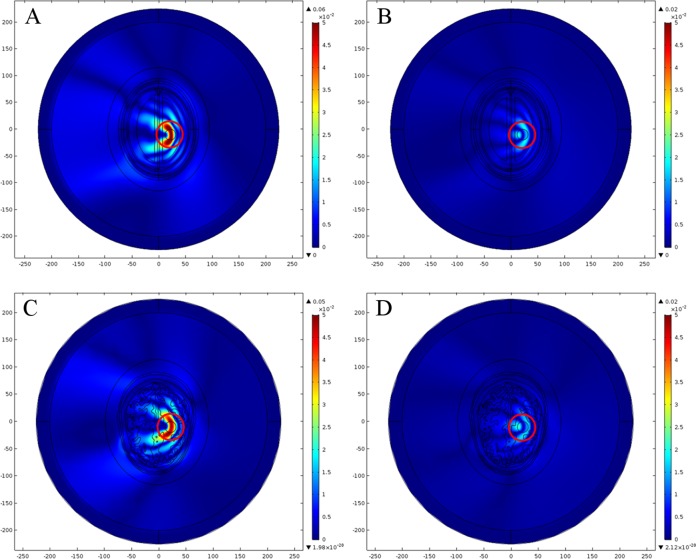
Spatial distribution of E-Norm absolute difference between the normal and stroke affected head models simulated at location 1 and 1 GHz frequency. (A) Ellipse head model with hemorrhagic stroke. (B) Ellipse head model with ischemic stroke. (C) Realistic head model with hemorrhagic stroke. (D) Realistic head model with ischemic stroke.

The analyses of E-Norm absolute difference for both head models were performed with each type of stroke emulated at a simple location 1. The point source 1 operating at 1 mA current and 1 GHz frequency was utilized in each scenario. Figure 18 shows the spatial distribution of E-Norm absolute difference between the normal and stroke affected head models, emulated at a simple location 1. In figure, the color bar represents E-Norm absolute difference (V/m) with red color indicating the locations of greater absolute differences and the blue color highlights the lower ones. Table 11 provides the details of E-Norm absolute difference between the normal and stroke affected head models evaluated at location 1. The simulations highlighted that the maximum E-Norm absolute difference existed at an approximate location of stroke in each case (encircled red).

**Table 11:** E-Norm absolute difference (V/m) between the normal and stroke affected head models evaluated at location 1 using Pt Src1 at 1 GHz frequency.

Head model	Stroke type	*Q3* (V/m)	*Q*4 (V/m)	Center (V/m)	*Q*2 (V/m)	*Q*1 (V/m)
**Ellipse model**	Normal	0.19556	0.21699	0.22545	0.22885	0.27305
	Hemorrhagic	0.19288	0.1622	0.17665	0.17558	0.26013
	Ischemic	0.17654	0.22568	0.22256	0.23974	0.2879
	Abs diff normal to hem	0.00268	0.05479	0.0488	0.05327	0.01292
	Abs diff normal to isch	0.01902	0.00869	0.00289	0.01089	0.01485
**Realistic model**	Normal	0.18699	0.23623	0.22742	0.22481	0.29296
	Hemorrhagic	0.19083	0.18752	0.18078	0.1751	0.26876
	Ischemic	0.16554	0.2454	0.22316	0.22969	0.30899
	Abs diff normal to hem	0.00384	0.04871	0.04664	0.04971	0.0242
	Abs diff normal to isch	0.02145	0.00917	0.00426	0.00488	0.01603

#### Brain stroke at complex location 2

**Figure 19:**
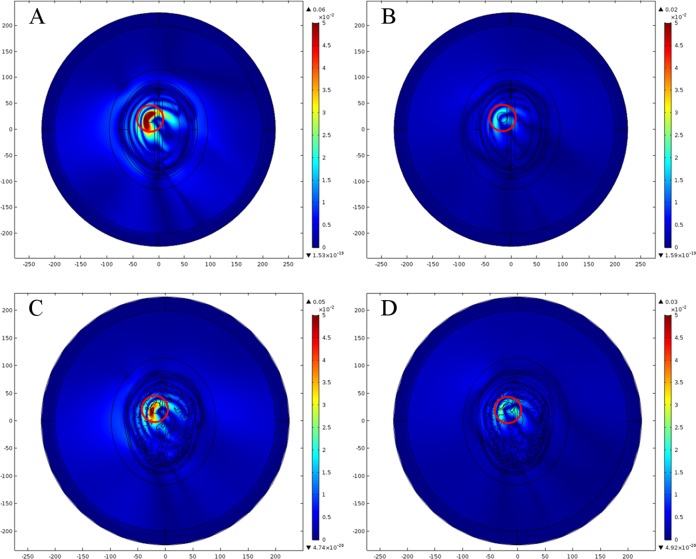
Spatial distribution of E-Norm absolute difference between the normal and stroke affected head models simulated at location 2 and 1 GHz frequency. (A) Ellipse head model with hemorrhagic stroke. (B) Ellipse head model with ischemic stroke. (C) Realistic head model with hemorrhagic stroke. (D) Realistic head model with ischemic stroke.

Similar analyses were performed for both head models with each type of stroke (hemorrhagic and ischemic) but emulated at a complex location 2. The point source 7 operating at 1 mA current and 1 GHz frequency was utilized in each scenario. Figure 19 shows the spatial distribution of E-Norm absolute difference between the normal and stroke affected head models, emulated at a complex location 2. Table 12 provides the details of E-Norm absolute difference between the normal and stroke affected head models evaluated at location 2. Similar to the above case, the maximum E-Norm absolute difference also existed at an approximate location of stroke in each scenario (encircled red).

**Table 12:** E-Norm absolute difference (V/m) between the normal and stroke affected head models evaluated at location 2 using Pt Src7 at 1 GHz frequency.

Head model	Stroke type	*Q*3 (V/m)	*Q*4 (V/m)	Center (V/m)	*Q*2 (V/m)	*Q*1 (V/m)
**Ellipse model**	Normal	0.26592	0.2185	0.24836	0.2812	0.135
	Hemorrhagic	0.21712	0.1883	0.19805	0.2382	0.11828
	Ischemic	0.2858	0.20782	0.24334	0.29997	0.12383
	Abs diff normal to hem	0.0488	0.0302	0.05031	0.043	0.01672
	Abs diff normal to isch	0.01988	0.01068	0.00502	0.01877	0.01117
**Realistic model**	Normal	0.2675	0.2026	0.206	0.2442	0.10706
	Hemorrhagic	0.23094	0.18641	0.17446	0.21352	0.10702
	Ischemic	0.28831	0.19039	0.20471	0.26137	0.090462
	Abs diff normal to hem	0.03656	0.01619	0.03154	0.03068	0.00004
	Abs diff normal to isch	0.02081	0.01221	0.00129	0.01717	0.016598

It has been observed that the maximum E-Norm absolute difference values are higher in the case of hemorrhagic stroke once compared to the ischemic stroke. It is due to the fact that dielectric properties of the hemorrhagic affected tissues (ε_r_ = 61.065, σ = 1.5829 S/m) contrast reasonably with respect to white matter tissues (ε_r_ = 38.577, σ = 0.6219 S/m). In comparison, dielectric properties of the ischemic affected tissues (ε_r_ = 30, σ = 0.5 S/m) are much closer to white matter tissues. It is worth mentioning that in each case the major part of stroke is emulated deep inside the white matter region too. Therefore, in the case of the hemorrhagic stroke, microwave signals experience more scattering at the stroke location once compared with ischemic stroke. These results ascertain the feasibility of microwave imaging for the identification and classification of different types of brain stroke. We also compared our simulation studies on microwave scattering behavior of the normal and stroke affected head models with latest research papers (Ireland & Abbosh, 2013; Mustafa, Abbosh & Nguyen, 2014; Nguyen, Mustafa & Abbosh, 2013). It is noteworthy to mention that our results were found in good agreement with their concluding facts and figures. In addition, we preferred FEM numerical method over FDTD to solve the subject microwave scattering problem in frequency domain and perform the frequency-swept analysis in a time-efficient manner.

It is important to understand that 2-D modeling does not fully reflect the anatomically realistic results of a complete head microwave scattering phenomenon. However, it serves as a time-efficient and computationally inexpensive method for the prediction of 3-D modeling and analysis. This is significant to address before we proceed to the complex 3-D simulations. The 2-D head modeling and microwave scattering analysis established in this study can be used as a quick indicator as to how a 2-D modeling approach may be extrapolated to 3-D realistic analysis. Therefore, it is realized that 3-D head modeling and simulations would provide more realistic results of microwave scattering phenomenon exhibited by the human head. In future, we are looking forward to develop an anatomically realistic complete head model and a wideband antenna array to perform 3-D microwave scattering analysis for brain stroke detection.

In recent years, numerous research groups have performed 3-D computer simulations and case studies on possible detection of brain stroke. The literature will exclusively guide us during our future research on 3-D microwave head imaging using anthropomorphic human head models. Khorshidi et al. (2009) demonstrated the efficacy of using a subspace distance based classifier to differentiate bleeding stroke patients from the non-bleeding ones. The potential performance of 3-D UWB-Magnitude Combined tomographic algorithm during the multi-frequency differential brain imaging was investigated by Guardiola et al. (2012). Wang, Al-Jumaily & Simpkin (2013) studied the application of 3-D holographic microwave and aperture synthesis imaging technique for brain stroke detection in a 3-D ellipsoid head model. Petrovic, Otterskog & Risman (2015) designed a contacting waveguide applicator for the detection of brain anomalies using microwave signals. Later on, the authors developed a magnetic field applicator for the visualization of brain hemorrhages using diffraction phenomenon (Petrovic, Otterskog & Risman, 2016). A wearable head imaging system comprising an array of directional monopole antennas was also proposed (Bashri, Arslan & Zhou, 2017).

Since, the microwave head imaging requires finding out the solution of an inverse scattering problem to develop an efficient image reconstruction algorithm. Therefore, the information about E-Field differences obtained from the forward problem solution may be fully exploited during this development. The inverse scattering problem is a mathematically non-linear and ill-posed problem, solved for a large number of unknowns. The developed image reconstruction algorithm extracts the dielectric properties of brain tissues utilizing only the information of transmitted and scattered E-Field signals. The calculated dielectric profile is mapped to create a high quality brain images and identify the areas of abnormal tissues.

Based upon the application in hand, multiple non-linear inversion methods are being employed in microwave imaging field. We performed a literature based analysis to compare the computational performance and accuracy of various inversion methods implemented in microwave head imaging studies. The most common in practice are; Gauss–Newton inversion (GNI), contrast source inversion (CSI), confocal delay-and-sum algorithm and born iterative method (BIM) (Ireland, Bialkowski & Abbosh, 2013; Ireland & Bialkowski, 2011; Mohammed et al., 2014; Mustafa, Mohammed & Abbosh, 2013; Semenov et al., 2007; Zakaria, Gilmore & LoVetri, 2010). We envisage that the CSI or multiplicative regularized CSI (MR-CSI) is more suitable to solve a microwave head imaging inverse problem. Because, this inversion method does not depend on the forward solution recalls during multiple iterations (Zakaria, Gilmore & LoVetri, 2010; Zakaria, Jeffrey & LoVetri, 2013). Therefore, in our future studies we will develop an image reconstruction algorithm based on CSI inversion scheme, by making use of the information generated from our present study’s forward problem solution.

### Specific absorption rate (SAR) analysis

In order to ensure the safe exposure of microwave signals to human head tissues, we also performed a SAR analysis. The SAR value determines the amount of radiations absorbed by a human body tissue and a temperature increase suffered under the exposure of electromagnetic signals. In earlier research studies, the SAR values for human head tissues were calculated using mobile phones. Most of them were based on computer simulations involving the numerical head models (Abdulrazzaq & Aziz, 2013; Citkaya & Seker, 2012; Morega & Morega, 2011; Wessapan, Srisawatdhisukul & Rattanadecho, 2012); and some covered the theoretical analysis as well (Sallomi, 2012). SAR is defined as the amount of power dissipated per unit mass and calculated using Eq. (3) in watts per kilogram [W/kg]. In Eq. (3), σ is the tissue electric conductivity [S/m], *E* is the induced electric field intensity norm value [V/m] and ρ is the tissue density [kg/m^3^]. We performed a SAR analysis for both types of head model to compare the ionizing effects of microwave signals in each case. Four point source locations (1, 5, 9 and 13) were utilized during these evaluations. Each point source was operating at 1 mA current with 1 GHz frequency and placed at a 2–3 cm distance from the side of head model.

(3) $${\bi{SAR}} = {{\rsigma {E^2}} \over {2\rho }}$$

**Figure 20:**
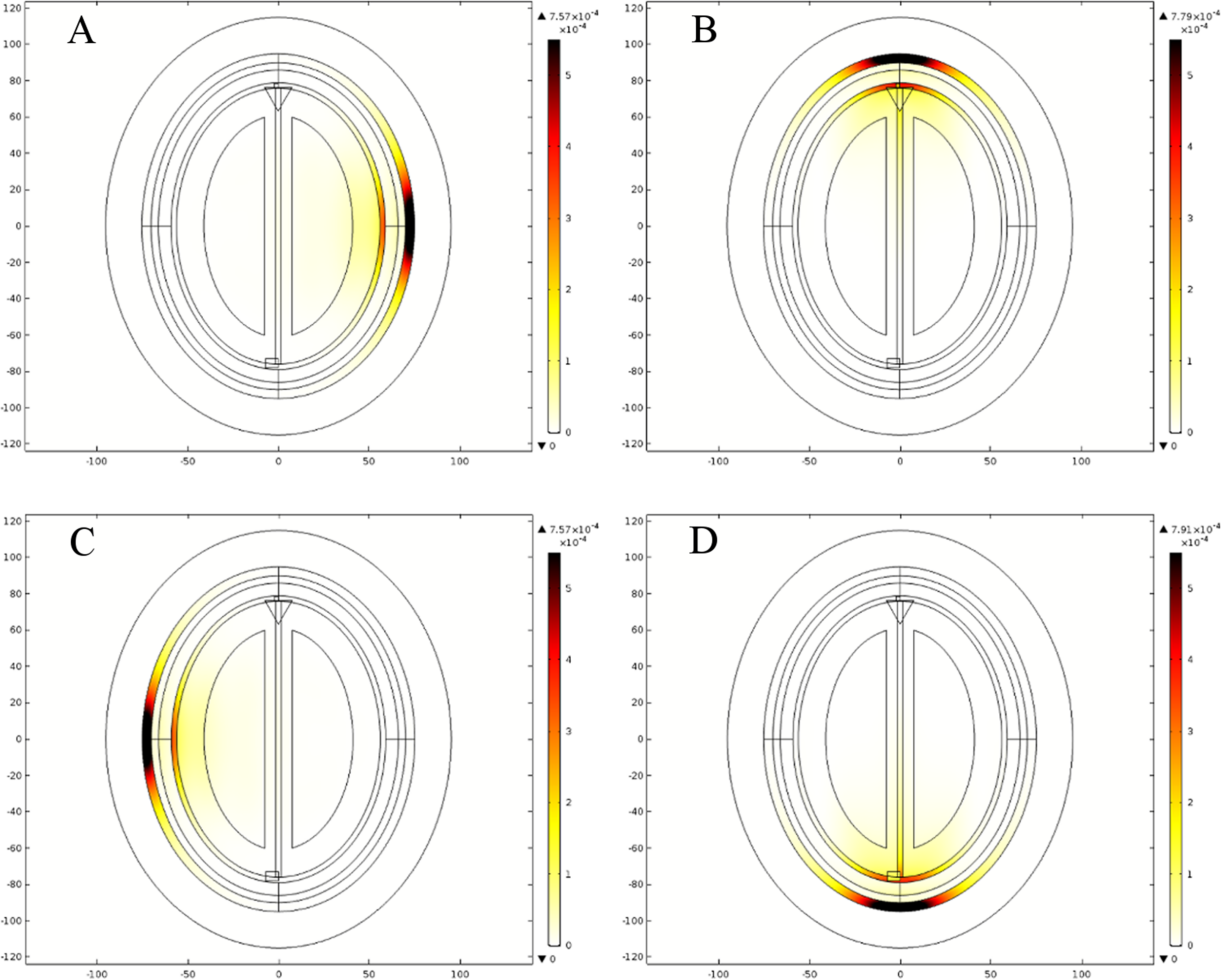
Spatial distribution of SAR value (W/kg) inside the ellipse head model at 1 GHz frequency. (A) Point source 1. (B) Point source 5. (C) Point source 9. (D) Point source 13.

**Figure 21:**
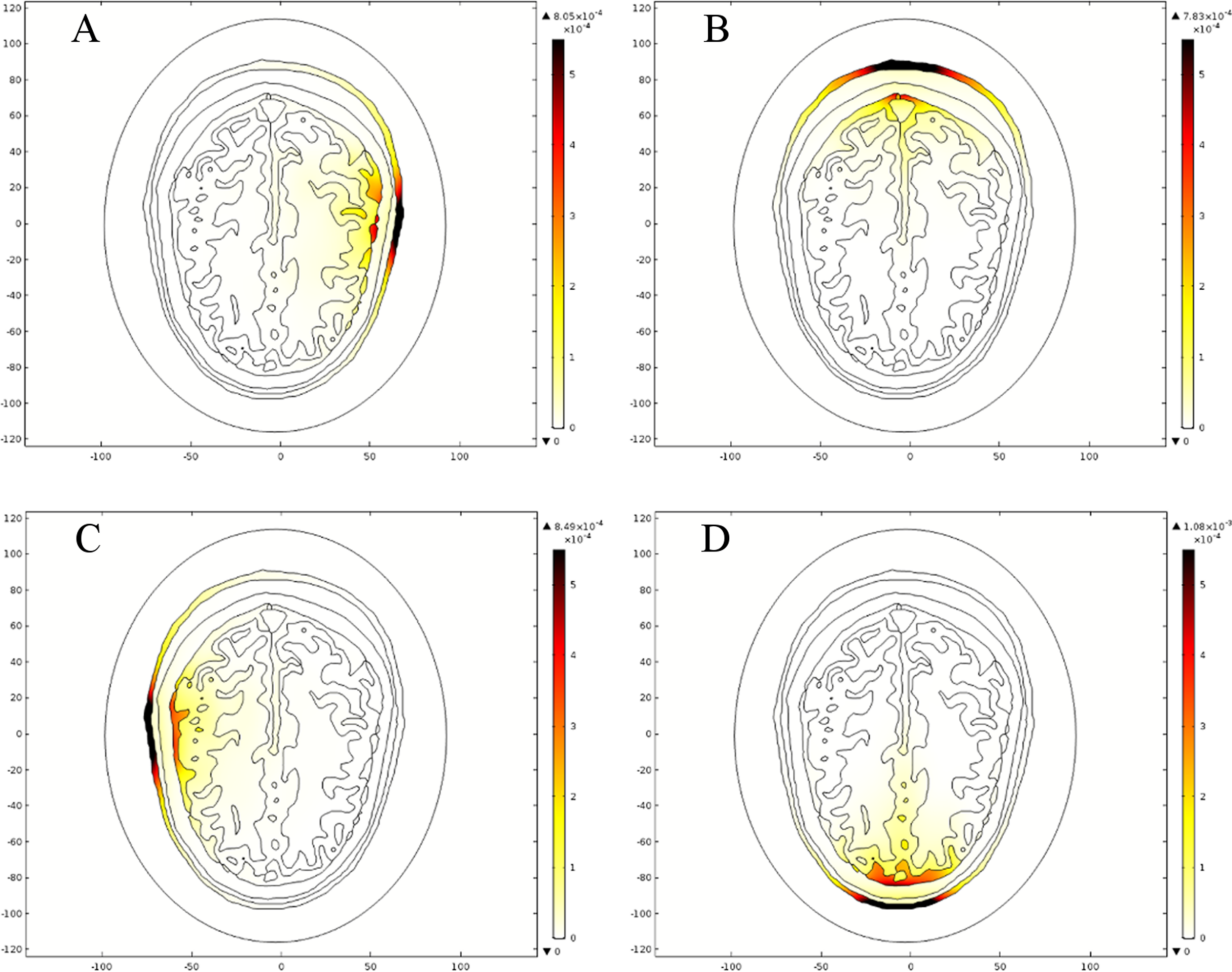
Spatial distribution of SAR value (W/kg) inside the realistic head model at 1 GHz frequency. (A) Point source 1. (B) Point source 5. (C) Point source 9. (D) Point source 13.

For both head models, the maximum local SAR value was observed for a point source positioned in front of the head at location 13. In the case of the ellipse head model, the maximum local SAR value was 7.91e^−4^ W/kg, whereas it was 1.08e^−3^ W/kg in the case of the realistic head model. Figures 20 and 21 shows the spatial distribution of SAR inside the ellipse and the realistic head models for four different point sources, respectively. In figures, the thermal color bar represents local SAR value (W/Kg) with reddish brown color indicating the locations of higher SAR values and the white color highlights the lower ones. It is pertinent to highlight that these values are far below the safety limit of average SAR (2 W/kg over 10 g of tissue) as per ICNIRP non-ionizing radiation protection guidelines (ICNIRP, 1998) and IEEE C95.1-2005 EM safety standard (IEEE, 2006). It has also been observed that the maximum local SAR value and the values at the interface of each layer of brain tissues are very close to each other in both types of head model (Table 13). Moreover, the maximum local SAR value is evaluated at the skin layer and it is highly dependent on the conductivity of brain tissues as the microwave signals travel into the head models.

**Table 13:** SAR values (W/kg) across different layers of brain tissue evaluated using Pt Src1 at 1 GHz frequency.

Head model	WM	GM	CSF	Skull	Fat	Skin
**Ellipse model**	4.99E-05	2.01E-04	1.31E-04	3.93E-05	3.10E-04	5.04E-04
**Realistic model**	4.02E-05	2.34E-04	2.49E-04	4.14E-05	3.24E-04	3.90E-04
**Abs diff (W/kg)**	9.72E-06	3.28E-05	1.18E-04	2.09E-06	1.43E-05	1.15E-04

We also compared our SAR analysis results with the earlier research study (Mobashsher et al., 2016a). It was conducted on the Zubal head phantom using a directional antenna transmitting between 1.1 and 3.2 GHz frequency at 0 dBm power level. The authors reported the peak SAR value of 16e^−3^ W/kg at 1.8 GHz frequency, once a directional antenna was positioned in front of the head phantom. The SAR value at 1.1 GHz frequency was calculated as 6e^−3^ W/kg. All these values were lower than the safety limit criteria of 2 W/kg over 10 g of tissue (ICNIRP, 1998; IEEE, 2006). In our realistic head model simulations, we evaluated the peak SAR value of 1.08e^−3^ W/kg at 1 GHz frequency with a point current source (1 mA) positioned at the front location 13. It is worth mentioning that our SAR calculations approximately matched the results of previous studies. In addition, during our literature review we also determined that a microwave source transmitting between 0.5 and 4.5 GHz frequency at 0–20 dBm power level and placed at a 2–4 cm distance from the surface of human head, would be appropriate for the design of a safe microwave head imaging system with minimum ionizing effects.

## Conclusion

This paper has presented a comparative analysis on the effects of incorporating different levels of detail into human head models on the microwave scattering phenomenon. It has been demonstrated that as we increase the complexity of head models the microwave scattering behavior contrasts considerably. The contrast between same locations inside both types of head models (i.e., geometrically simple and anatomically realistic) is greater in a complex region once compared to the simpler one. Finite element method is adopted to solve the subject microwave scattering problem due to its flexibility to model complex geometry of the human head with least discretization error. Using FEM, the solution for E-Field values against varying levels of mesh density converged to a reasonable value as well.

It has been shown that the subject microwave scattering problem can be efficiently solved using either the PARDISO direct solver or the FGMRES iterative solver. Since it is a 2-D problem the PARDISO direct solver is recommended to solve such problems in a computationally time-efficient manner. The simulation results verified that the microwave imaging may successfully highlight the location of different types of brain stroke using a difference between E-Field values of the normal and the stroke affected brain tissues. This information may effectively be utilized to develop an efficient image reconstruction algorithm based upon the calculation of dielectric properties of brain tissues, to detect the position and types of brain stroke, which is our future paradigm too. During SAR analysis, it has been observed that the maximum local SAR value and the values across different layers of brain tissue are very close to each other in both types of head model and within the safety limit as well.

In future, we will present a microwave scattering analysis of the 3-D human head models using various levels of detail. The simulation results of the present 2-D microwave scattering problems will be utilized to develop an efficient image reconstruction algorithm using CSI inversion method. In order to realize the real-life conditions, the noise will be added to the simulated E-Field database and its effects on the results of image reconstruction algorithm will be investigated too. The algorithm will be made robust against the noise effects using an appropriate noise cancellation technique. We will also implement the frequency sweeping in our simulations to analyze the frequency-dispersive behavior of microwave scattering from the human head models. This multi-frequency swept approach may also be employed to construct a better quality brain images in a recursive method. In order to achieve a high-performance solution, the parallel processing techniques will be considered during the multi-frequency multi-source implementation in the microwave scattering forward problem simulations and the image reconstruction algorithm development.

## Supplemental Information

10.7717/peerj.4061/supp-1 Supplemental Information 1 Raw data and code.

## Additional Information and Declarations

### Data Availability

The following information was supplied regarding data availability: The viewable data and code files have been attached as Supplemental Files.
